# A novel tailed primer nucleic acid test for detection of HPV 16, 18 and 45 DNA at the point of care

**DOI:** 10.1038/s41598-023-47582-y

**Published:** 2023-11-21

**Authors:** Megan M. Chang, Ariel Ma, Emilie Newsham Novak, Maria Barra, Kathryn A. Kundrod, Jane Richards Montealegre, Michael E. Scheurer, Philip E. Castle, Kathleen Schmeler, Rebecca Richards-Kortum

**Affiliations:** 1 Department of Bioengineering, Rice University, Houston, TX USA; 2 Division of Cancer Epidemiology and Genetics, National Cancer Institute, Rockville, MD USA; 3 Department of Behavioral Science, The University of Texas MD Anderson Cancer Center, Houston, TX USA; 4 Department of Pediatrics Hematology/Oncology, Baylor College of Medicine, Texas Children’s Hospital, Houston, TX USA; 5 Division of Cancer Prevention, National Cancer Institute, Rockville, MD USA; 6 Department of Gynecologic Oncology and Reproductive Medicine, The University of Texas MD Anderson Cancer Center, Houston, TX USA

**Keywords:** Cancer screening, Biomedical engineering

## Abstract

Cervical cancer is a leading cause of death for women in low-resource settings despite being preventable through human papillomavirus (HPV) vaccination, early detection, and treatment of precancerous lesions. The World Health Organization recommends high-risk HPV (hrHPV) as the preferred cervical cancer screening strategy, which is difficult to implement in low-resource settings due to high costs, reliance on centralized laboratory infrastructure, and long sample-to-answer times. To help meet the need for rapid, low-cost, and decentralized cervical cancer screening, we developed tailed primer isothermal amplification and lateral flow detection assays for HPV16, HPV18, and HPV45 DNA. We translated these assays into a self-contained cartridge to achieve multiplexed detection of three hrHPV genotypes in a disposable cartridge. The developed test achieves clinically relevant limits of detection of 50–500 copies per reaction with extracted genomic DNA from HPV-positive cells. Finally, we performed sample-to-answer testing with direct lysates of HPV-negative and HPV-positive cell lines and demonstrated consistent detection of HPV16, HPV18, and HPV45 with 5000–50,000 cells/mL in < 35 min. With additional optimization to improve cartridge reliability, incorporation of additional hrHPV types, and validation with clinical samples, the assay could serve as a point-of-care HPV DNA test that improves access to cervical cancer screening in low-resource settings.

## Introduction

The World Health Organization (WHO) has put out a call to action to accelerate the elimination of cervical cancer as a public health problem^1^. Although cervical cancer is preventable with human papillomavirus (HPV) vaccination, along with early detection and treatment of precancerous lesions, an estimated 341,000 women die from cervical cancer annually^2^. Nearly 90% of all cervical cancers and cervical cancer deaths occur in low- and middle- income countries (LMICs)^3,4^ due to the lack of access to high quality preventive services, which are challenging to implement and sustain in low-resource settings^5^.

The WHO recommends high-risk HPV (hrHPV) DNA testing as the preferred cervical cancer screening method due to its high sensitivity to detect cervical cancer and its precursors. hrHPV DNA testing has been shown to be more effective than screening with visual inspection with acetic acid (VIA) or cytology^6–11^. In LMICs, hrHPV DNA testing can be implemented as part of screen-and-treat programs, which allow women who screen positive for hrHPV to be treated in the same visit^5^.

Traditional methods of detecting hrHPV DNA, such as with hybrid capture or polymerase chain reaction (PCR), require expensive instruments, trained personnel, and reliable infrastructure, which are often inaccessible in LMICs. Although there are commercially available platforms for hrHPV testing designed for use in low-resource settings, they remain too costly and complex to support rapid, decentralized testing at the point of care. careHPV, a hybrid capture test developed for use in low-resource settings, requires expensive equipment and laboratory technicians to perform the testing. Furthermore, careHPV necessitates batching of samples to achieve the target per-test cost of $5 USD^12,13^, which delays test results, increasing the likelihood that patients will be lost to follow-up. GeneXpert HPV, another HPV test marketed for point-of-care use, has a high per-test cost of approximately $15 USD and requires costly instrumentation, limiting widespread adoption in LMICs^14^. Thus, a low-cost hrHPV test that can be performed at or near the point of care with rapid time to results could support the implementation and scaling of cervical cancer screening globally.

To meet this need, we present novel tailed primer recombinase polymerase amplification (RPA) and lateral flow detection assays for HPV16, HPV18, and HPV45 DNA. We used existing commercially available HPV DNA assays to inform the design criteria for this test. We benchmarked the desired sensitivity using digene HC2, which can detect 100,000 copies of hrHPV per mL of sample, or 100 copies of hrHPV per μL of sample^15^. Since RPA uses 10 μL of sample per reaction, this corresponds to a desired sensitivity of 1000 input copies per reaction. We designed a multiplexed lateral flow readout to provide partial genotyping capabilities (HPV16, 18/45) that is consistent with the WHO cervical cancer screening guidelines^5^ and that is similar to that of GeneXpert HPV, which reports separate results for HPV16 and HPV18/HPV45 positivity. We employed a qPCR-enabled quantitative tailed primer screening methodology and demonstrate its utility to develop sensitive and specific assays. Finally, we simultaneously performed these assays in a single tube with a custom insert and integrated amplification and lateral flow detection in an innovative, self-contained, disposable cartridge. We performed sample-to-answer testing on a field-deployable, commercially manufacturable platform (NATflow, Axxin Pty Ltd, Australia) with direct lysates from HPV-negative and HPV-positive cell lines and show that the developed test achieves a clinically relevant limit of detection with HPV-positive cells in less than 35 min.

## Results

To circumvent the complexity of traditional nucleic acid tests, we pursued an approach that couples isothermal amplification using RPA with lateral flow detection of amplicons. We employed a tailed primer amplification and lateral flow strategy, in which one of the primers contains a unique 15 base pair tail on the 5’ end that enables capture of amplicons with oligonucleotide hybridization on the lateral flow strip^16^, while the other primer contains a biotin label for visualization of amplicons through a traditional lateral flow sandwich assay using streptavidin labelled gold nanoshells. This approach is highly amenable to multiplexed readouts, as altering the tail sequence allows for capture of amplicons at separate test lines. However, tailed primer assays with RPA are prone to false positive results due to the lack of a sequence specific probe^17^ and hybridization specificity challenges due to RPA’s low operating temperature. Thus, to design sensitive and specific tailed primer assays for HPV16, HPV18, and HPV45, we implemented a quantitative primer screening methodology that identifies and quantifies specific and nonspecific RPA product formation using qPCR and melt curve analysis of qPCR products^18^.

Here, we describe results in which we first utilized this method to screen candidate untailed RPA Basic primers to identify primer pairs with high sensitivity and specificity. We then designed candidate tailed primer pairs and screened them with both this quantitative methodology and lateral flow analysis of tailed primer products to select sensitive and specific tailed primer assays. We subsequently evaluated the limits of detection (LoDs) with extracted genomic DNA from HPV-positive cell lines. Finally, we integrated these assays onto a sample-to-answer platform and assessed its analytic performance using both extracted cellular DNA as well as unpurified cell lysates from HPV-positive and HPV-negative cell lines in a simple sample-to-answer workflow.

### RPA basic primer screens

We designed untailed RPA Basic primers to amplify the E7 gene of HPV16, HPV18, and HPV45, and confirmed genotype specificity in silico using NCBI BLAST. We evaluated the sensitivity of primers by amplifying positive controls containing 50 or 100 input copies of short synthetic gene fragments containing the target gene, and the specificity of primers by amplifying a no-target gDNA control that is representative of an HPV-negative sample. This initial primer screen used input concentrations well below the target LoD of 1000 input copies per reaction^15^, as we hypothesized that the addition of a tail and transitioning to full-length targets could result in reduced sensitivity. RPA products were diluted and amplified in qPCR with the same RPA primer pair, and a positive qPCR control using target HPV DNA was included to identify specific product formation. Both specific and nonspecific amplification products were then identified and quantified.

**Figure 1:**
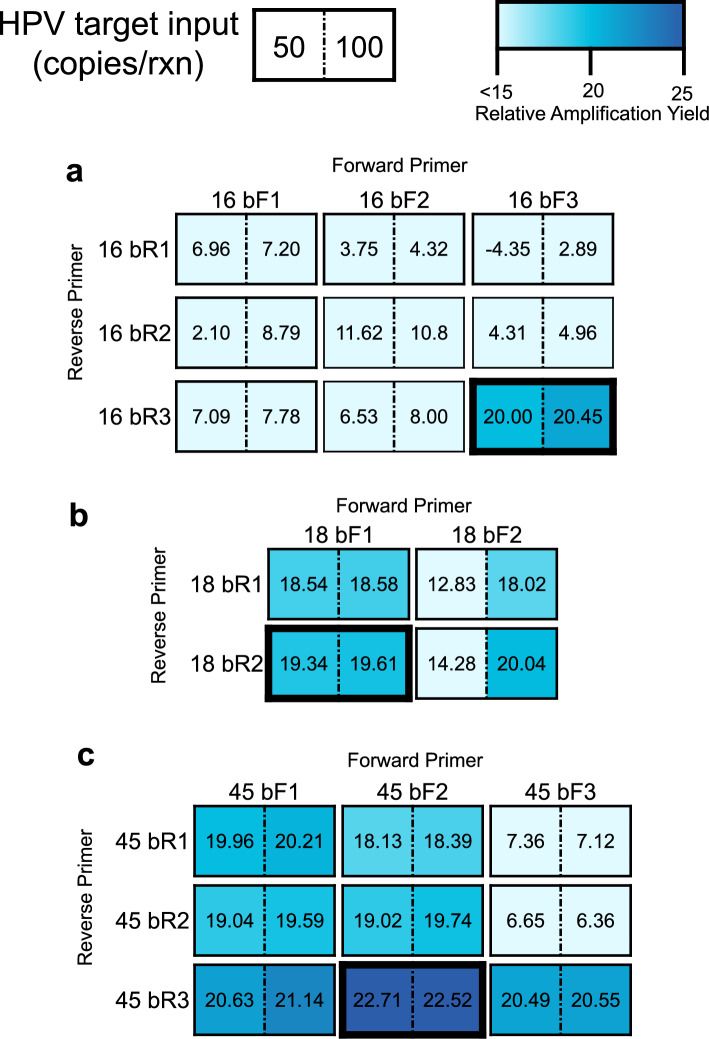
RPA Basic Primer Screening and Optimization. Target HPV gBlock DNA and no-target human genomic DNA were first amplified in RPA, and the resultant amplicons were diluted and analyzed in qPCR using the same primer pair as for the RPA reaction. The relative amplification yield of positive reactions, containing 50 or 100 input copies of target HPV DNA, to no-target reactions is displayed for primer pairs specific to (**a**) HPV16, (**b**) HPV18 and (**c**) HPV45. Data represent the mean relative amplification yield for nine replicates (three RPA replicates x three technical replicates). Outlined boxes indicate primer pairs with the greatest relative amplification yield for both 50 and 100 input copies of target HPV DNA that were chosen for tailed primer design.

The results of the initial primer screens are displayed in Fig. 1. We found that all the RPA reactions with no-target gDNA controls resulted in nonspecific products that amplified in qPCR, as confirmed by melt curve analysis (Supplementary Fig. S1, Fig. S2, Fig. S3). However, there were primer pairs for which these nonspecific RPA products amplified much later than RPA reactions containing the HPV target. To identify primer pairs that minimized this nonspecific product formation, which could lead to false positive lateral flow results in the absence of a sequence specific probe^17^, we calculated the relative amplification yield of RPA reactions containing positive controls compared to that of RPA reactions containing no-target gDNA, and selected primer pairs for HPV16, HPV18, and HPV45 that had the greatest relative amplification yield for further assay development. For HPV18 and HPV45, two closely related high-risk genotypes, we also experimentally validated that the predicted in silico genotype specificity was achieved (data not shown).

### RPA tailed primer screens

**Figure 2:**
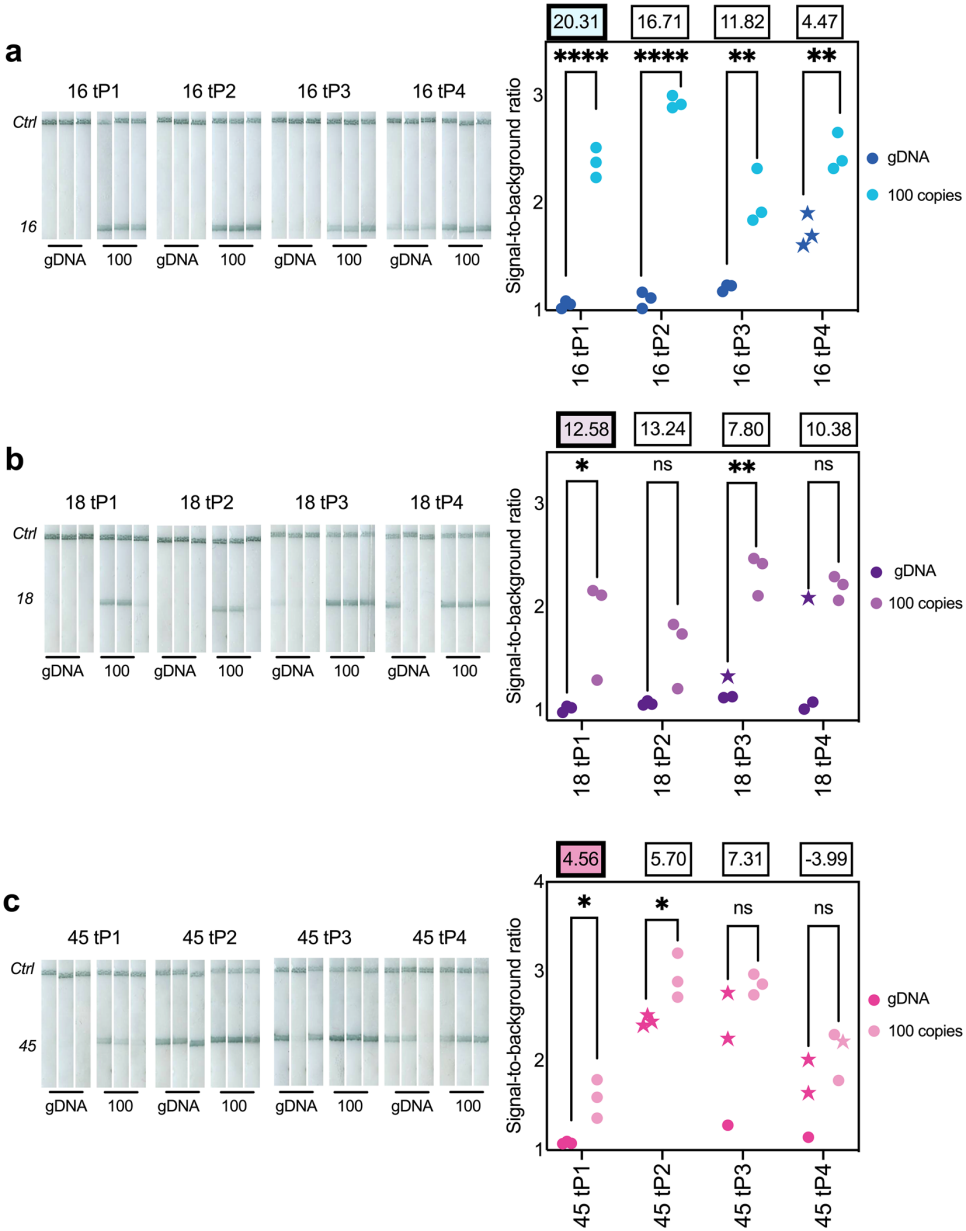
RPA tailed primer screens. Lateral flow strips (left) of RPA products from tailed primers designed for (**a**) HPV16, (**b**) HPV18, and (**c**) HPV45. Signal-to-background ratio (SBR) from each strip (right) and mean relative amplification yield (n = 9; three RPA replicates x three technical replicates) are displayed above each primer pair. Starred replicates indicate nonspecific products, confirmed by melt curve analysis, which produced a positive visual signal. Tailed primer pairs chosen for further assay development are highlighted. *p < 0.05; **p < 0.01; ***p < 0.001; ****p < *0.0001; ns* = *not significant; significance determined for each primer pair by a two-tailed unpaired t-test*.

After identifying primer pairs with the highest relative amplification yield, we then designed four sets of tailed primer pairs for each target HPV genotype and measured their performance by quantifying reaction yields in qPCR and analyzing products with custom lateral flow devices (Supplementary Fig. S4). Results of these tailed primer screens are shown in Fig. 2. From these tailed primer screens, we identified tailed primer pairs for HPV16 (16 tP1), HPV18 (18 tP1), and HPV45 (45 tP1) for further assay development. All these pairs had no visible signal generated on the lateral flow strips by no-target gDNA controls and achieved a statistically significant difference at the 0.05 significance level in signal-to-background ratios between negative and positive reactions on the lateral flow strips. In cases where more than one tailed primer pair met the above criteria (16 tP1-3), the tailed primer pair with the highest relative amplification yield was chosen.

Of note, we observed that despite efforts to choose primer pairs that minimized nonspecific primer dimer amplification products in the initial screening, and designing tailed primer pairs that did not result in any additional heterodimerization, many of the tailed primer pairs resulted in false positive results on lateral flow strips, which were confirmed by melt curve analysis to be the result of nonspecific amplification products (Supplementary Fig. S5, Fig. S6, Fig. S7). Moreover, most tailed primer pairs had a lower relative amplification yield with 100 input copies of gBlock DNA than the original nontailed primer pair. For example, HPV45 only had one primer pair (45 tP1) that had no signal generation with no-target gDNA controls, and had a markedly reduced relative amplification yield compared to the best performing tailed primer pair for HPV16 or HPV18. We hypothesize that nonspecific product formation, indicated by the presence of a small product peak around 70 °C in addition to the target product peak at 80 °C (Supplementary Fig. S7) is responsible for this reduced amplification yield.

### Limit of detection with extracted cellular DNA

After designing tailed primer assays that were specific and able to amplify short gene fragments at the desired LoD, we sought to evaluate the tailed primer assays with more complex targets. We extracted cellular DNA from SiHa (HPV16-positive), HeLa (HPV18-positive) and MS751 (HPV45-positive) cell lines and tested a range of input concentrations of this extracted cellular DNA with the tailed primer amplification and detection assays.

**Figure 3:**
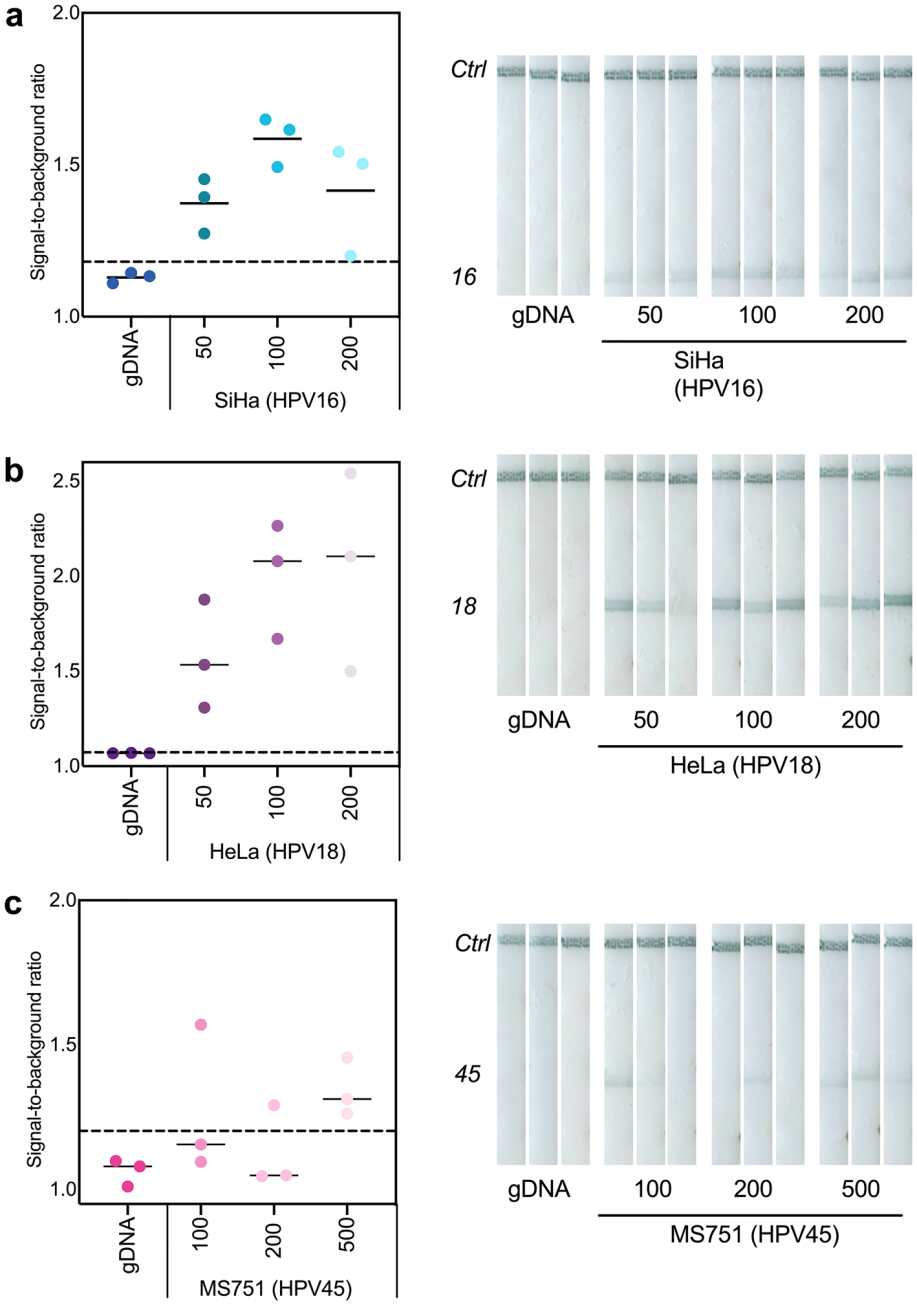
Tailed primer assays achieve sensitive detection with cellular extracts from HPV-positive cells. No-target human gDNA and extracted DNA from (**a**) SiHa cells (HPV16), (**b**) HeLa cells (HPV18) and (**c**) MS751 cells (HPV45) were amplified with tailed primer assays and detected on lateral flow strips. SBR of each replicate (left) and lateral flow strips in triplicate (right) are displayed for each type-specific assay.

We found that the HPV16 and HPV18 tailed primer assays could detect 50 copies per reaction of SiHa and HeLa DNA, respectively (Fig. 3a and b). The LoD for the HPV45 tailed assay was an order of magnitude higher at 500 copies per reaction (Fig. 3b); this reduction in sensitivity for the HPV45 tailed primer assay is likely due to the reduced relative amplification yield seen in the tailed primer screens. However, this LoD is still below the predefined benchmark^15^.

### Integrated amplification and detection in NATflow cartridge

**Figure 4:**
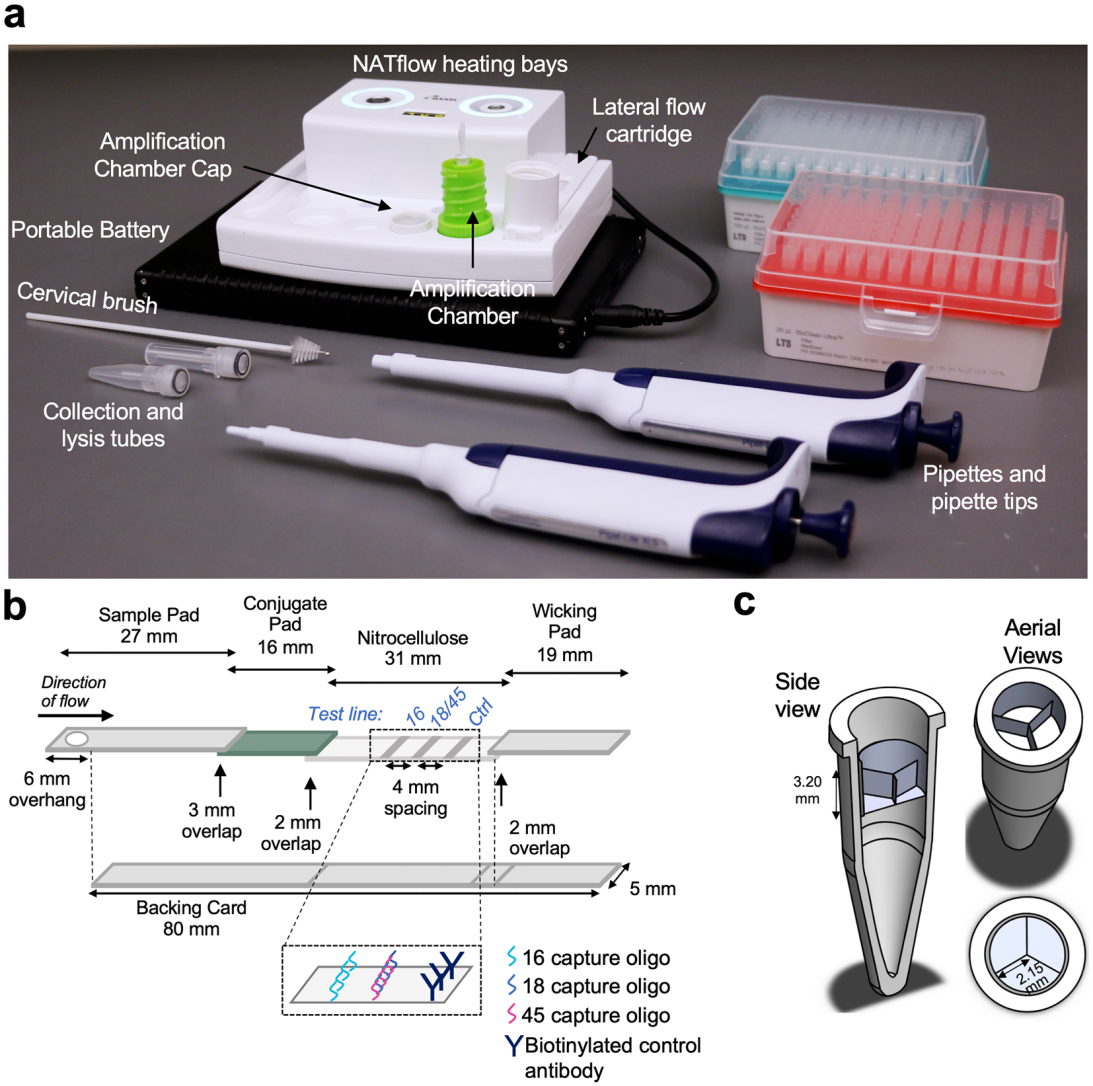
NATflow overview. (**a**) NATflow instrument and associated consumables needed to run the tailed primer HPV assay. (**b**) Schematic of NATflow-compatible lateral flow strip. Lateral flow strips for integrated testing on the NATflow were modified from^19^ to decrease running time, reduce strip-to-strip variability, and increase manufacturability at scale. (**c**) Custom PCR tube insert for multiplexed testing on the NATflow. The insert fits inside a high-profile PCR tube and divides the reaction space into three wells that each hold up to 15 *µ*L. The insert allows reactions for HPV16, HPV18, and HPV45 to be spatially separated in a single NATflow cartridge, thus reducing cost and complexity associated with running multiple reactions simultaneously.

We then implemented the tailed primer assays in a sample-to-answer platform suitable for point-of-care testing (Fig. 4; NATflow, Axxin Pty Ltd). The NATflow platform consists of an instrument with two programmable heating bays, and a disposable cartridge that integrates isothermal amplification and lateral flow detection that is fully enclosed to prevent workspace contamination with amplified DNA^19^ (Fig. 4a). The cartridge has three components: 1) an amplification chamber, which contains a PCR tube for the reaction to occur; 2) an elution buffer cap, which snaps atop the amplification chamber to prevent aerosolization of amplicons during incubation and contains the necessary running buffer for amplicon dilution and lateral flow visualization of products on custom lateral flow strips (Fig. 4b); and 3) a lateral flow cartridge base, which contains a plunger that pierces the elution buffer cap and displaces liquid in the amplification chamber to deliver it to the sample pad of the lateral flow strip in the cartridge base. For amplification to occur, the amplification chamber is placed into a heating bay. After incubation, the amplification chamber is inverted and twisted into the lateral flow cartridge base for amplicon dilution and lateral flow visualization of products. The cartridge can then be interpreted visually or by an optional lateral flow reader (AX-2X-S, Axxin Pty Ltd).

To circumvent the challenges of biochemically multiplexing the three tailed primer assays into a single RPA reaction, which can result in loss of sensitivity^20^, we developed a custom PCR-tube insert that spatially separates the three tailed primer assays within the integrated NATflow cartridge (Fig. 4c) while maintaining compatibility with the elution mechanism of the cartridge. During the elution step, when the amplification chamber is twisted into the lateral flow cartridge, the plunger in the base of the lateral flow cartridge collapses the insert to deliver the products of each amplification reaction to the sample pad of the lateral flow strip. We validated that the insert could successfully deliver fluid from all three chambers to the sample pad of the lateral flow strip in the NATflow cartridge (Supplementary Fig. S8), and optimized heating parameters of the NATflow heating bay to achieve a reaction temperature of 39 °C within the insert (Supplementary Fig. S9).

### Performance of integrated assays with extracted genomic DNA from HPV-positive cell lines

**Figure 5:**
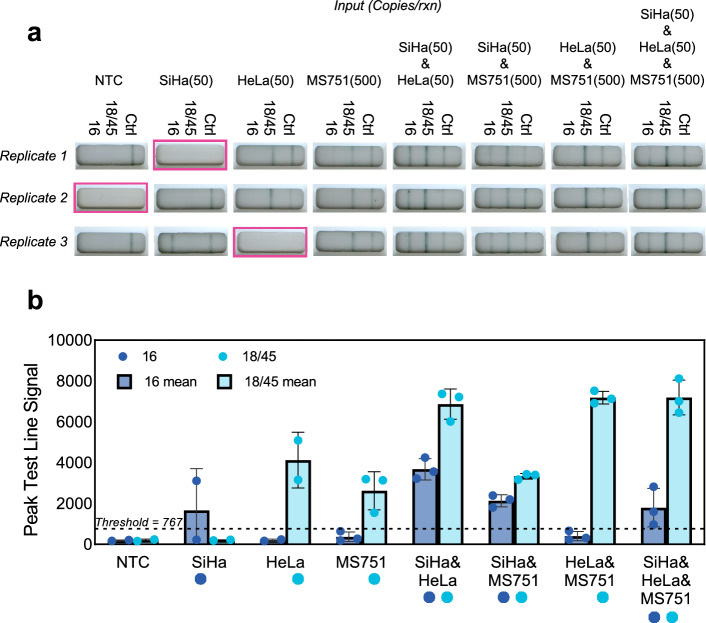
Integrated cartridge performance with custom PCR insert. (**a**) Reactions containing gDNA or extracted cellular DNA from SiHa (HPV16-positive), HeLa (HPV18-positive), and/or MS751 (HPV45-positive) cells at the indicated input concentrations were run in triplicate in the NATflow cartridges with the custom PCR insert. Highlighted images indicate invalid tests due to cartridge failures that were excluded from analysis. (**b**) Mean ± standard deviation of the signal generation at test lines, as determined by the AX-2X-S lateral flow reader of the replicates shown in (**a**). Circles represent individual replicates, and circles beneath the x-axis indicate expected 16 or 18/45 positivity. Signal above the positivity threshold (dashed line) is only seen at test lines when the target HPV genotype is present.

We evaluated performance of assays in the NATflow cartridge using extracted genomic DNA from HPV-positive cell lines at the previously determined LoDs (from Fig. 3) using inputs that contained one (singleplexed) or more (multiplexed) target HPV types. Results are shown in Fig. 5. Three of 24 cartridges had lateral flow strip failures where no test or control line signal formed; these cartridges were considered invalid and excluded from subsequent analysis. Of the tests with valid results, we found that sensitivity and specificity of the tailed primer assays were maintained with both single- and multi-plexed inputs in this integrated format. With 50 input copies of extracted SiHa DNA, the HPV16 tailed primer assay resulted in one of two replicates with signal generation above the positivity threshold with SiHa as a singleplexed input. When extracted SiHa DNA was multiplexed with other targets, all nine replicates correctly generated positivity at the 16 test line above the positivity threshold. Similarly, the HPV18 and HPV45 tailed primer assays also resulted in sensitive detection at the previously determined LoDs of 50 or 500 copies with extracted HeLa and MS751 DNA, respectively, in both singleplexed and multiplexed detection. All valid cartridges containing 50 input copies of extracted HeLa DNA and/or 500 copies of extracted MS751 DNA resulted in signal generation at the 18/45 test line above the positivity threshold. Moreover, we found that the tailed primer assays were also specific in this integrated multiplexed format, as signal above the positivity threshold was only seen at test lines when the corresponding target HPV genotype was present. There was a faint signal at the off-target HPV16 test line when MS751 (HPV45) DNA was amplified as a singleplexed input, but the signal is below the positivity threshold.

### Sample-to-answer testing with HPV-negative and HPV-positive cell lines

**Figure 6:**
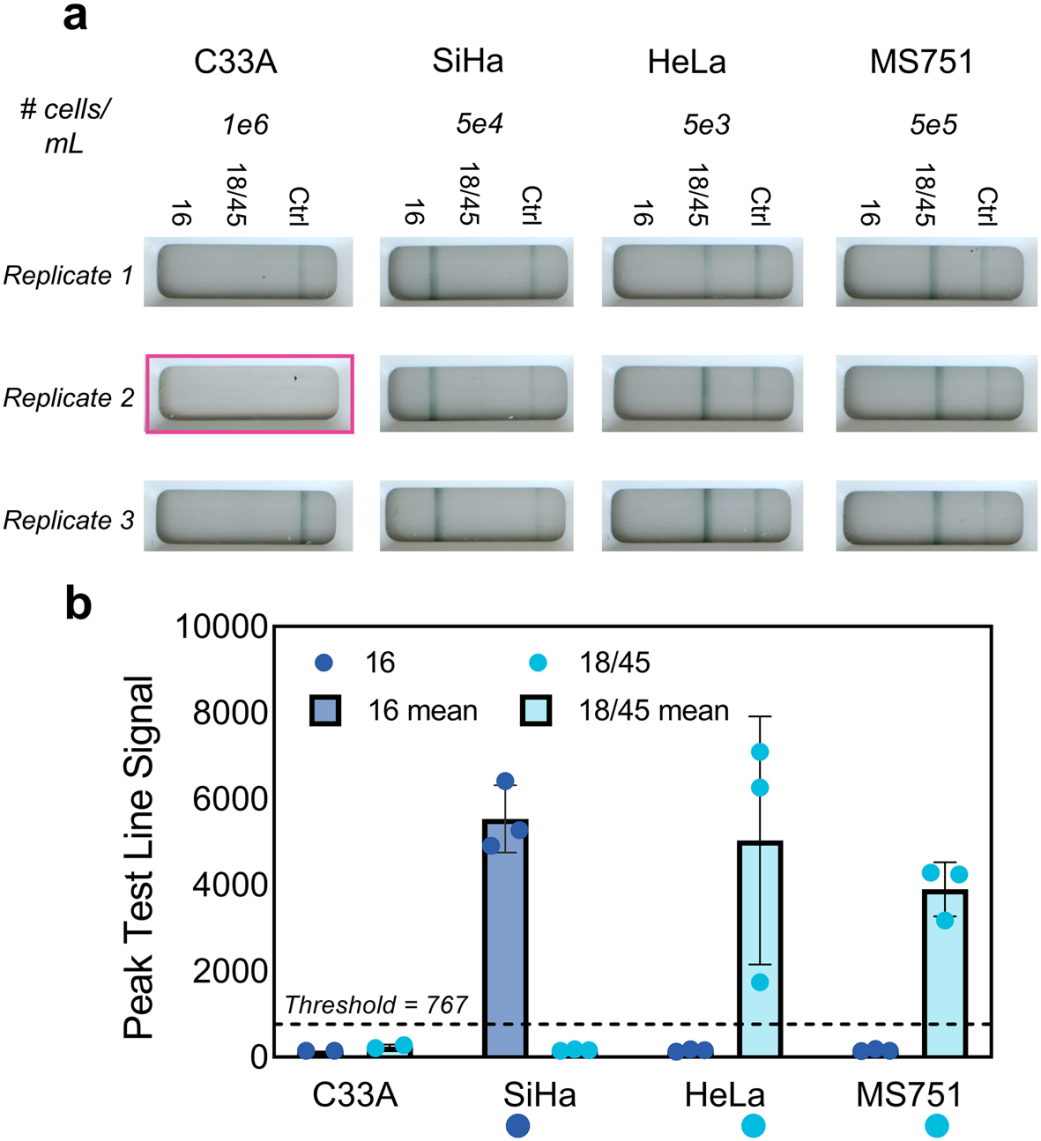
Sample-to-answer testing with HPV-negative and HPV-positive cells. (**a**) C33A (HPV-negative), SiHa (HPV16-positive), HeLa (HPV18-positive), or MS751 (HPV45-positive) cells at the indicated concentrations were processed with a point-of-care compatible ACP-based sample preparation protocol, and the resultant lysates were amplified on NATflow instrumentation and detected in the integrated NATflow cartridge. Replicate cartridge images are shown (n = 3), with the highlighted cartridge image indicating an invalid test due to lack of fluid flow down the lateral flow strip. (**b**) Mean ± standard deviation of the peak test line signal intensity, as determined by the AX-2X-S lateral flow reader of the replicates shown in (a). Circles represent individual replicates, and circles beneath the x-axis indicate expected 16 or 18/45 positivity. All cellular lysates generated strong visible signal at the target HPV test line above the positivity threshold (dashed line) and had clean signals at the off-target test lines. C33A resulted in no signal generation at either the HPV16 or the HPV18/45 test line.

Finally, we demonstrated proof-of-concept sample-to-answer testing capabilities using HPV-negative (C33A) and HPV-positive cellular samples at clinically relevant concentrations. Cellular samples were prepared at either 5e3 cells/mL, 5e4 cells/mL or 5e5 cells/mL, and combined with a lysis buffer containing ACP enzyme. Samples were incubated at room temperature for five minutes, and then at 95 °C for five minutes to deactivate the ACP enzyme^19,21^. Cellular lysate was then directly input into the HPV16, HPV18 and HPV45 tailed primer amplification assays. Amplification and lateral flow detection was carried out on the NATflow platform with the custom PCR insert, and the resultant cartridges are displayed in Fig. 6, with only one invalid cartridge.

In this sample-to-answer testing demonstration, we found that there was reliable detection for all target HPV types from cellular lysates, and no-off target amplification of lysate from C33A, an HPV-negative cell line. There was strong, consistent signal generation at the HPV16 test line using SiHa cells at 5e4 cells/mL. SiHa cells contain 1–2 copies of HPV16 per cell^22^, thus the theoretical amount of HPV16 DNA in SiHa lysate, assuming a 90% ACP lysis efficiency^23^, is 88 input copies of HPV 16 (Eq. 1).

1 $$\begin{aligned} & 5*10^{4} \frac{SiHa\,cells}{{{\text{mL}}}} \times \frac{{1 {\text{mL}}}}{{1000 {\mu L}}} \times \frac{{1 {\text{HPV16}}\,copy}}{cell} \times \frac{{196 {\mu L} \,cells}}{{200 {\mu L} \,lysate}} \\ & \quad \times 90\% \, lysis\, efficiency \times \frac{{2 {\mu L} \,lysate}}{{10 {\mu L} \,reaction}} = 88 \,input\, copies\, {\text{HPV16}} \,{\text{DNA}} \\ \end{aligned}$$

Similarly, there was strong amplification and detection of HeLa lysate resulting from HeLa cells at a concentration of 5e3 cells/mL. This increased sensitivity with HeLa is expected, as HeLa contains 10–50 copies of HPV18 DNA per cell^22^; thus at 5e3 cells/mL, the theoretical amount of HPV18 DNA from HeLa lysate is also 88 input copies (Eq. 2).

2 $$\begin{aligned} & 5*10^{3} \frac{HeLa \,cells}{{{\text{mL}}}} \times \frac{{1{\text{ mL}}}}{{1000 {\mu L}}} \times \frac{{10 \,{\text{HPV18}}\, copies}}{cell} \times \frac{{196 {\mu L}\, cells}}{{200 {\mu L} \,lysate}} \\ & \quad \times 90\% \, lysis \,efficiency \times \frac{{2 {\mu L} \,lysate}}{{10 {\mu L}\, reaction}} = 88\, input\, copies \,{\text{HPV18}} \,{\text{DNA}} \\ \end{aligned}$$

Finally, strong amplification of MS751 lysate was observed using a concentration of 5e5 cells/mL, which corresponds to a theoretical input of 882 copies of HPV45 DNA per reaction (Eq. 3), since MS751 cells have 1–2 copies of HPV45 DNA per cell^24,25^. The increased amount of MS751 cells is also consistent with previous experiments, where the HPV45 tailed primer assay is an order of magnitude less sensitive than the HPV16 or HPV18 tailed primer assays.

3 $$\begin{aligned} & 5*10^{5} \frac{MS751 \,cells}{{mL}} \times \frac{1mL}{{1000 \mu L}} \times \frac{1 HPV45\,copy}{{cell}} \times \frac{196 \mu L\,cells}{{200 \mu L\, lysate}} \\ & \quad \times 90\% \,lysis \,efficiency \times \frac{2 \mu L\,lysate}{{10 \mu L \,reaction}} = 882\, input\,copies \,HPV45 \,DNA \\ \end{aligned}$$

## Discussion

We successfully developed sensitive and specific tailed primer amplification and lateral flow detection assays for HPV16, HPV18, and HPV45 DNA. We spatially multiplexed these tailed primer assays into a novel cartridge that is disposable, self-contained, and manufacturable. We demonstrated proof-of-concept, sample-to-answer testing with HPV-negative and HPV-positive cellular samples, with results available in less than 35 min.

To develop this test, we adapted a quantitative primer screening methodology^18^ to enable the identification and quantification of both specific and nonspecific RPA products. Tailed primer assays are prone to false positive results due to the lack of a sequence specific probe^17^, thus we utilized this quantitative methodology to identify primer pairs that minimized the formation of nonspecific amplification products resulting from primer dimerization, which could not be predicted with thermodynamics alone. Although there are tools to help with RPA primer design^26^, RPA assays often require extensive screening and optimization^27^. The primer screening methodology presented here could permit a more streamlined workflow and accelerate the primer screening and optimization process.

When evaluated with extracted genomic DNA from HPV-positive cells, the tailed primer assays were found to have a LoD of 50 copies per reaction of HPV16 and HPV18 DNA and 500 copies per reaction of HPV45 DNA. These LoDs are more sensitive than the benchmark of digene HC2^15^.The HPV45 tailed primer assay is less sensitive than the developed HPV16 and HPV18 assays, likely due to some off-target primer-dimerization products, but is still below the benchmarked sensitivity. It is important to note that the goal of HPV test development is not to develop an ultrasensitive assay, but rather to develop an assay calibrated to identify patients likely to have CIN2 + disease. Indeed, the benchmarked LoD of 100,000 copies per mL has been shown to optimize the clinical sensitivity and specificity of a screening test^28^, and assays that detect low viral load infections lead to increased HPV positivity without an increase in clinical sensitivity^29^. At the same time, it is important to recognize that the sensitivity of many isothermal nucleic acid tests decreases as the complexity of the sample increases (i.e. with transition from extracted DNA, to unpurified cellular lysates, to lysates of clinical samples)^19,30–33^.

We integrated isothermal amplification and lateral flow detection of the three tailed primer assays into a disposable cartridge. A custom PCR insert was developed to spatially separate the three reactions within the same tube, while still maintaining compatibility with the cartridge elution mechanism. This strategy allowed the assays to maintain a sensitive LoD with extracted genomic DNA in the integrated cartridge format. Previous studies with the NATflow cartridge have demonstrated slightly reduced sensitivity after integration^19^. Finally, a previously developed point-of-care sample preparation protocol using ACP enzymatic lysis and heat deactivation was incorporated into the test^19,21^, and sample-to-answer testing was demonstrated with unpurified lysates from HPV-negative and HPV-positive cell lines. Results demonstrate that sensitive detection is maintained with cellular lysates using input concentrations close to the LoD. This proof-of-concept demonstration is a first step toward direct point-of-care testing of cervical swabs collected into non-preservative TE buffer; however, cultured cells lack potential inhibitors such as mucus or blood that could impact assay performance when testing with patient samples^34^. Clinical validation of the developed test is needed to assess the sensitivity and specificity of the test in the presence of potential inhibitors.

The developed assay and cartridge are low cost and additional cost reductions are expected with at-scale manufacturing. The assay has a per-test reagent cost of ~ $9 USD (Table S5) and an instrumentation cost of $500 USD for the NATflow heater. This is more affordable than GeneXpert, which has an estimated per-test cost of approximately $15 USD^14^, and an instrument cost of approximately $5000 USD for the portable, battery-powered, single module Omni model^35^. Moreover, in contrast to careHPV, the per-test cost can be achieved without batching samples. The most expensive reagent of the current assay is the conjugate reporter, gold nanoshells; alternative reporters such as latex^36^ or gold nanoparticles^20^ could be used instead to further reduce per-test reagent costs. Further, in comparison to other isothermal amplification tests developed for hrHPV DNA detection using lateral flow^37–39^ or fluorescence readouts^40,41^, our integrated test is designed to be self-contained and user-friendly without the need for dedicated optical instrumentation.

Additional optimization is needed before the PCR insert can be reliably incorporated into a point-of-care test. In testing both extracted cellular DNA and cellular lysates in the integrated format, there was an overall cartridge failure rate of 11%, likely due to mechanical failures introduced by the insert that led to incomplete displacement of elution buffer from the amplification chamber cap. In addition, the plunger mechanism of the NATflow lateral flow cartridge sometimes split the PCR tube open upon during elution due to the pressure of the crushed plastic insert against the side of the tube. This opening of the amplification chamber to the environment could lead to workspace contamination with amplified DNA and subsequent false positive results. Further testing with various materials is needed to reduce the probability of invalid tests and contamination. Finally, the PCR insert still requires manual reaction assembly and pipetting of small volume reactions into millimeter-sized wells. The development of a user-friendly sample transfer device, such as a modified dropper bottle, to reliably deliver sample into the wells, and the incorporation of lyophilized small volume RPA reactions into the insert^19^, could circumvent these challenging user steps.

While HPV16, HPV18 and HPV45 account for ~ 77% of invasive cervical cancers^42^, recent research indicates that an effective cervical cancer screening test needs to incorporate a total of eight hrHPV genotypes (16, 18, 31, 33, 35, 45, 52, and 58) that account for ~ 90% of all cervical cancers is needed in order to maximize the clinical sensitivity and specificity of detecting cervical cancer and precancer^43,44^. With additional primer design, the quantitative screening methodology presented here could extend to the development of additional tailed primer assays, and biochemical multiplexing of these tailed primers could be achieved with careful optimization. Previous studies have multiplexed amplification of up to three DNA targets in a single reaction^17,45,46^, and in combination with the PCR insert described here, could permit detection of eight hrHPV genotypes and a cellular control in a single cartridge. In addition, the high specificity of oligonucleotide capture demonstrated with our results could allow for separate readout in an array format^47^, thus enabling extended genotyping capabilities in a low-cost format. However, an extended genotyping test would likely require an external reader to read multiple lines or an array, thus increasing user complexity and instrumentation costs. We envision that the tailed primer approach could potentially support both a screen-and-treat model and a screen-triage-treat model given its partial genotyping capabilities^5^, and that other hrHPV types could be incorporated at a third test line.

In conclusion, we developed a simple sample-to-answer test utilizing tailed primer isothermal amplification and lateral flow visualization of amplified products to detect DNA from HPV16, HPV18 and HPV45. This initial study of sample-to-answer testing with HPV-negative and HPV-positive cells illustrates the potential of the developed test to provide sensitive and rapid detection of hrHPV from cervical samples. With further optimization, inclusion of additional hrHPV types, and validation with patient samples, the developed test could enable rapid hrHPV testing at the point of care, an important step towards scaling up and increasing access to screen-and-treat programs in low-resource settings.

## Methods

### Cell culture

C33A (HPV-negative), SiHa (HPV16-positive), HeLa (HPV18-positive), and MS751 (HP-45 positive) cells were acquired from the American Type Culture Collection (Manassas, VA). Cells were passaged up to ten times, pelleted in quantities of 0.5–15 million cells, and stored at  − 80 °C until use.

### Clean reaction setup

Amplification reaction setup was conducted inside a separate pre-amplification room. A dedicated biosafety cabinet was used for all DNA extraction and target preparation. All pre-amplification and sample preparation spaces and equipment were regularly decontaminated with bleach, RNase Away, and/or UV light. Single-use nuclease-free water aliquots were used for both target dilution and reaction set-up. Post-amplification analysis of amplicons was conducted in a space physically separated from pre-amplification activities to prevent workspace contamination with amplified DNA. Each lab space (sample preparation, pre-amplification, post-amplification) had distinct lab coats to further mitigate the risks of environmental contamination with amplicons.

### Target DNA preparation

gBlocks Gene Fragment (gBlocks) DNA for the full E7 genes of HPV16 (Genbank ID K02718), 18 (Genbank ID X05015), and 45 (Genbank ID X74479) were purchased from Integrated DNA Technologies (Coralville, IA) and resuspended in 1X TE buffer. DNA from SiHa, HeLa, and MS751 cells was extracted using the DNeasy Blood and Tissue Kit (Qiagen) per manufacturer’s instructions, with a final elution into nuclease-free water. HPV16, HPV18, and HPV45 gBlock and cellular extract DNA were quantified with digital droplet PCR as described below. Single-use aliquots were prepared and stored at  − 80 °C for up to six months prior to use. Target DNA dilutions were prepared in nuclease-free water on each day of experiments.

Human genomic DNA (G3041, Promega, Madison, WI) was used as a no-target control in all RPA experiments. DNA was quantified by a NanoDrop 1000 Spectrophotometer, stored at  − 80 °C, and diluted to a concentration of 100 copies per μL in nuclease-free water before use.

### Digital droplet PCR

Primers for HPV16, HPV18 and HPV45 (Table 3.S1) were designed to amplify the E7 gene and purchased from IDT. 25 μL ddPCR reactions containing 1 × ddPCR EvaGreen Supermix (BioRad, Hercules, CA), 100 nM each of forward and reverse primers, 5 μL water and 6.25 μL template were assembled, of which 20 μL were transferred to the sample wells of a DG8 Cartridge (BioRad). 70 μL of Droplet Generation Oil for EvaGreen was loaded into the oil wells, and the DG8 cartridge was covered with a DG8 gasket and loaded into the QX200 Droplet Generator (BioRad) to generate ddPCR droplets. After droplet generation, ddPCR droplets were transferred to a clean 96-well plate, which was sealed with a foil heat seal using the PX1 PCR Plate Sealer (BioRad). Thermocycling was performed on a BioRad CFX-96 instrument according to manufacturer’s instructions with the heated lid set to 105 °C, the sample volume set at 40 μL, and a temperature ramp rate of 2 °C/sec. Reactions were thermocycled using the following steps: 95 °C enzyme activation for 5 min, 40 cycles of 95 °C denaturation for 30 s and annealing/extension at 60 °C (HPV16 and HPV 18) or 57 °C (HPV45) for 1 min, signal stabilization for 5 min at 4 °C then 5 min at 90 °C, and finally a 4 °C hold until reactions could be read. Fluorescence signals of each reaction were quantified by a QX200 Droplet Reader and quantified with QuantaSoft*™* software.

### RPA basic primer screens

Untailed RPA Basic primers were first designed using PrimedRPA^26^ and ordered from Integrated DNA Technologies (Coralville, IA). RPA Basic primers were screened using a no-target human gDNA control, and positive controls containing either 50 or 100 input copies of target HPV gBlock per reaction to identify primer pairs that resulted in strong amplification below the desired LoD and minimized the formation of nonspecific amplification products in the absence of HPV target DNA. After amplification, RPA products were immediately diluted 1:200 in nuclease-free water and analyzed with qPCR using the same primer pair as for RPA amplification.

Amplification curves and melt curves resulting from qPCR amplification of RPA products were used to identify and quantify nonspecific and specific product formation. A positive qPCR control containing 5000 input copies of gBlock target was included for each primer pair, and its melt curve was used to differentiate specific products from primer dimerization products. The amplification yield of each reaction was calculated by subtracting the reaction’s Ct value from the average Ct value of the qPCR positive control [reaction yield = Ct (positive control)—Ct (reaction)]. Positive control RPA reactions resulting in the formation of nonspecific products, determined by melt curve analysis, were assigned an amplification yield of zero.

Finally, the relative amplification yields for each primer pair were calculated by subtracting the average reaction yield of the no-target gDNA control from the average reaction yield of the positive controls [relative amplification yield = average reaction yield (positive control)—average reaction yield (no-target gDNA control)]. The primer pair with the highest relative amplification yield for both 50 and 100 copies was selected for tailed primer design.

### Tailed primer screens

Promising RPA Basic primer pairs for HPV16, HPV18, and HPV45 were then used to design tailed primers. Tailed primers contain a unique 15 base pair barcode at the 5’ end that allows for lateral flow capture of amplicons^16^. Tailed primers were designed using a custom MATLAB script (Supplemental Code File S1) that randomly generated unique 15 base pair sequences and appended them to the 5’ end of the original RPA Basic primer. These tailed primers were then analyzed using MATLAB’s Bioinformatics Toolbox^48^ and filtered so that the final tailed primer sequences: (i) had an AT rich tail (GC content less than 45%)^17,49^; (ii) resulted in a tailed primer with no additional self-dimerization or hairpins compared to the original untailed RPA Basic primer; and (iii) resulted in no additional cross-dimerization between the tailed primer pair compared to the untailed primer pair.

For each high-risk HPV type, four tailed primer pairs (Table S3) were screened by amplifying a no-target gDNA control and 100 copies of input gBlock DNA. Tailed primer amplification products were diluted 1:200, analyzed in qPCR, and relative amplification yields were calculated as described above. Additionally, amplicons were analyzed in a custom lateral flow device (Fig. S4) fabricated as described in *Lateral flow assays*. Following amplification with RPA, tailed products were diluted 1:50 in a running buffer containing 4X SSC, 1.4% Triton X-100, and 5% formamide^16^. 10 μL of diluted products was added to the sample pad, 20 μL of 2.5 OD streptavidin-coated gold nanoshells in 0.5% BSA in 1X PBST was added to the conjugate pad, 30 μL of running buffer was added to the buffer pad, and devices were folded to initiate flow.

### RPA

50 μL RPA Basic (TwistDx, Maidenhead, UK) reactions were assembled on cold blocks according to manufacturer’s instructions. Briefly, each enzyme pellet was rehydrated with a master mix containing 29.5 μL of rehydration buffer, 2.4 μL each of forward and reverse primers (10 μM in 1X TE), and 3.2 μL of nuclease-free water. After rehydration, the pellets were thoroughly mixed by pipetting up and down. 10 μL of target was added and mixed by pipetting. Finally, 2.5 μL of 280 mM magnesium acetate was included in the cap of each reaction tube. Reactions were initiated with a brief spin in a minicentrifuge, gently vortexed, and spun again before incubating on a T8-ISO for 20 min at 39 °C. A two mm stainless steel mixing ball (Simply Bearings Ltd, Manchester, UK), cleaned by washing in 1% SDS followed by thorough rinsing with nuclease-free water and two ethanol rinses, was dispensed into each reaction using a microball dispenser (TwistDx) to provide continual mixing during incubation.

### qPCR analysis of RPA products

qPCR reactions were prepared in 20 μL volumes using PowerUp SYBR Green Master Mix (Applied Biosystems). All reactions contained 10 μL of 2X Master Mix, 1 μL each of forward and reverse primer at 10 μM working concentrations, 3 μL of nuclease-free water, and 5 μL of sample. After sample addition, qPCR plates were sealed with optically clear MicroSeal ‘B’ PCR Plate Sealing Film (Bio-Rad), spun in a plate centrifuge for 15 s, then thermocycled on a Bio-Rad CFX96 for amplification and detection. Thermocycling was performed using standard cycling conditions according to manufacturer’s instructions, with annealing and extension occurring for 1 min at 60 °C, followed by a melt curve analysis.

### Lateral flow assays

All paper and plastic components for lateral flow assays were laser cut (Universal Laser Systems VLS 3.60, Scottsdale, AZ) and assembled by hand.

#### Lateral flow devices for tailed primer screens

Devices for tailed primer screens (Fig. S4) were constructed with clear adhesive-backed film (Blick Art Supplies 55525-1021, Galesburg, IL), 0.005″ clear plastic (Blick Art Supplies 55506-1005), UniStart CN95 nitrocellulose (Sartorius, Goeingen, Germany), cellulose (Millipore CFSP223000, Burlington, MA), and glass fiber (Ahlstrom 8951, Mt Holly Springs, PA). Capture oligonucleotides (Table S4), containing 60 base pairs of repeating reverse complements to primer tails, were ordered from IDT and used at 100 μM working concentrations. A sciFLEXARRAYER S3 (Scienion, Berlin, Germany) was used to print approximately 21.6 pmol of capture oligonucleotide per strip. Biotinylated goat anti-mouse control antibody, prepared as previously described^20^, was deposited at the control line. After drying at room temperature, test strips were exposed to UV light (UVP HL-2000 HybriLinker) at 125 mJ/cm^2^ for two minutes to crosslink the capture oligonucleotides to the nitrocellulose^50^ before being stored in a desiccated foil pouch until use.

#### NATflow-compatible strips

NATflow-compatible lateral flow strips (Fig. 4b) were produced using similar methods with the following modifications and stored at room temperature in a desiccated foil bag until use. A sciFLEXARRAYER S3 was used to deposit approximately 1.6 nmol of capture oligonucleotides and biotinylated anti-mouse IgG on a 31 mm × 250 mm backed sheet of nitrocellulose (Unistart CN95, Sartorius, Goeingen, Germany); the HPV16 line was printed 14.5 mm from the proximal edge, the HPV18/45 test line was offset 4 mm to the distal end, and the control line was offset an additional 4 mm from the HPV18/45 test line.

Conjugate pads were prepared by diluting streptavidin-coated gold nanoshells (GSIR150, Nanocomposix, San Diego, CA) in a three to five ratio with 5% BSA in 1X PBST. Two mL were pipetted onto a 16 mm × 250 mm glass fiber pad (Grade 8980, Ahlstrom, Mt Holly Springs, PA), then lyophilized without freezing for at least 24 h in a freeze-drying system (LabConco FreeZone 12, Kansas City, MO). After lyophilization, the conjugate pad was then used for lateral flow strip assembly.

Lateral flow strips were assembled on an 80 mm backing card (MIBA-010, Diagnostics Consulting Network, Carlsbad, CA). The covertape between the kiss cuts at 17 mm and 43 mm was removed, and the nitrocellulose membrane was aligned on the exposed adhesive backing. Then, a 19 mm × 250 mm cellulose wicking pad (CFSP223000, Millipore) was placed on the distal end of the nitrocellulose membrane with an overlap of two mm. The conjugate pad was placed on the proximal end of the nitrocellulose membrane, also overlapping by two mm. Finally, a 27 mm × 250 mm glass fiber sample pad (Ahlstrom Grade 8951) was placed at the proximal end, overlapping three mm with the conjugate pad with a six mm overhang. The assembled card was cut into five mm wide strips using an A-Point Digital guillotine cutter (Arista Biologicals, Allentown, PA, USA). Before placing into a NATflow cartridge, a 3.5 mm hole was punched (ASONTAO Leather Hole Punch A, Amazon) into the overhang, offset two mm from the proximal end of the strip.

### Integrated sample-to-answer testing on the NATflow

#### NATflow PCR insert fabrication

SOLIDWORKS© was used to create a cylindrical PCR insert with three chambers of 15 μL volume (Fig. 4c). PCR inserts were printed on a Formlabs Form 3B + printers with Formlabs Black Resin (Somerville, MA). After printing, the pieces were washed for 10 min in isopropyl alcohol in the Formlabs Form Wash and then cured for 30 min at 60 °C in the Formlabs Form Cure according to manufacturer’s instructions.

#### Lysis of cellular samples

Achromopeptidase (ACP) lysis buffer was prepared at a working concentration of 25 U/μL in 1X TE buffer. SiHa, HeLa, and MS751 cell pellets were reconstituted in 1X TE buffer and diluted to concentrations of 5e3 cells/mL to 5e5 cells/mL in the same buffer. 196 μL of the contrived cellular sample was combined with 4 μL of ACP lysis buffer to reach a final concentration of 0.5 U/μL ACP^21,23^. Samples were incubated at room temperature for 5 min to allow lysis to occur, then heated at 95 °C for 5 min to deactivate the ACP enzyme. Cell lysates were placed on ice until use.

#### NATflow cartridge assembly

NATflow amplification chambers and lateral flow cartridges were assembled on the same day of use. NATflow compatible lateral flow strips prepared as described above were placed into NATflow lateral flow cartridges. High-profile PCR tubes, provided by the manufacturer, were placed into amplification chambers, and the PCR insert was placed inside each tube. Elution buffer caps were loaded with 170 μL of running buffer containing 4X SSC, 1.4% Triton X-100, and 5% formamide.

#### Amplification and detection

HPV16, HPV18, and HPV45 tailed primer RPA assays were prepared as described above with either human gDNA, extracted cellular DNA, or cellular lysate as the target. Reactions were initiated with magnesium acetate catalyst before quickly aliquoting 10 μL of activated reaction mixture to each well of the PCR insert. Amplification chambers were capped with the elution buffer ring, then incubated on a NATflow heater set to 45 °C for 20 min. Following amplification, amplification chambers were inverted and twisted into the lateral flow cartridge to elute amplicons and running buffer onto the sample pad of the lateral flow strip. Cartridges were allowed to flow for 3 min before scanning on a lateral flow reader (AX-2X-S, Axxin Pty Ltd) and on a flatbed scanner (Epson Perfection V550).

### Image analysis

Signal-to-background ratios (SBR) at the test and control lines for strips run in the lateral flow devices were calculated using a custom image analysis program in MATLAB. Raw RGB images were converted to grayscale and inverted, and the mean pixel intensity values across the width of the strip were computed to create a mean intensity line profile along the length of the strip. The signal at the HPV16 and the HPV 18/45 lines was found by searching for the highest peaks within 15 pixel windows centered 70 or 50 pixels from the control line peak, respectively; if no peaks were found, then the median pixel value of the mean intensity line plot in each window was taken. The background value was found by taking the average of the highest peaks of the inverted mean intensity line profile, and the signal-to-background ratio was calculated by dividing the test line signal over the background signal.

For lateral flow strips run in NATflow cartridges, cartridge images obtained by the AX-2X-S lateral flow reader were processed using an algorithm developed by Axxin Pty Ltd^19^ to identify the control and test lines within the read window, and calculate the peak signal intensity at each line.

### Statistical analysis

To evaluate whether differences in signal generation between no-target gDNA controls and positive samples containing target HPV DNA for the tailed primer screens were statistically significant, a two-tailed unpaired t-test assuming equal variances was performed. To set positivity thresholds for the tailed primer LoD study, the SBR of three no-target gDNA controls was calculated, and the positivity threshold was set at the mean SBR signal of the no-target controls plus three standard deviations. To set positivity thresholds for lateral flow strips run in NATflow cartridges, the mean peak test signal at the 16 and combined 18/45 test line was averaged for no-target gDNA controls and samples containing off-target HPV genotypes, and the threshold was set three standard deviations above that average.

### Supplementary Information

Supplementary Information.

Supplementary Information.

## Data Availability

All data generated or analyzed during this study are included in this published article (and its Supplementary Files). The code used to generate tailed primer pairs is included as a Supplementary File.
