# Supplementary material for: A novel tailed primer nucleic acid test for detection of HPV 16, 18 and 45 DNA at the point of care
Source: Sci Rep. 2023 Nov 21;13:20397. doi: 10.1038/s41598-023-47582-y (PMC10663460; doi:10.1038/s41598-023-47582-y)
Supplement: Supplementary file 1 — Supplementary Information. [file 41598_2023_47582_MOESM1_ESM.docx]

# Supplementary Information for

A novel tailed primer nucleic acid test for detection of HPV 16, 18 and 45 DNA at the point of care

Megan M. Chang, Ariel Ma, Emilie Newsham Novak, Maria Barra, Kathryn A. Kundrod, Jane Richards Montealegre, Michael E Scheurer, Philip E. Castle, Kathleen Schmeler, Rebecca Richards-Kortum^*^

This supplementary information includes Figures S1-S9 and Tables S1-S5, which detail primer and capture probe sequences, melt curve analyses from primer screens, the custom NATflow insert validation, and cost estimates.

| Primer Name | Primer Sequence (5’🡪3’) |
| --- | --- |
| 16 qF10 | GCAACCAGAGACAACTGATCTC |
| 16 qR11 | GTGCCCATTAACAGGTCTTCC |
| 18 qF11 | GCAATTAAGCGACTCAGAGGAAG |
| 18 qR11 | GCTGCTGGAATGCTCGAAGG |
| 45 qF10 | CCGGGAAACACTGCAAGAAATT |
| 45 qR10 | CCGGGAAACACTGCAAGAAATT |

Supplementary Table S1: HPV ddPCR primers. All primers used in ddPCR to quantify HPV targets.

| Primer Name | Primer Sequence (5’ 🡪 3’) |
| --- | --- |
| 16 bF1 | CATGGAGATACACCTACATTGCATGAATAT |
| 16 bF2 | ATGGAGATACACCTACATTGCATGAATATA |
| 16 bF3 | CACCTACATTGCATGAATATATGTTAGATT |
| 16 bR1 | CACAACCGAAGCGTAGAGTCACACTTGCAA |
| 16 bR2 | GCACAACCGAAGCGTAGAGTCACACTTGCA |
| 16 bR3 | TAGTGTGCCCATTAACAGGTCTTCCAAAGT |
| 18 bF1 | ATTGCATTTAGAGCCCCAAAATGAAATTCC |
| 18 bF2 | AAGACATTGTATTGCATTTAGAGCCCCAAA |
| 18 bR1 | AGCTTTCTACTACTAGCTCAATTCTGGCTT |
| 18 bR2 | TACTAGCTCAATTCTGGCTTCACACTTACA |
| 45 bF1 | GCATTTGGAACCTCAGAATGAATTAGATCC |
| 45 bF2 | TGCATTTGGAACCTCAGAATGAATTAGATC |
| 45 bF3 | ATTGCATTTGGAACCTCAGAATGAATTAGA |
| 45 bR1 | AGCTCTCTACTGTAAGCTCAATTCTGCCGT |
| 45 bR2 | AGCTCTCTACTGTAAGCTCAATTCTGCCGT |
| 45 bR3 | TCTCTACTGTAAGCTCAATTCTGCCGTCAC |

Supplementary Table S2: RPA Basic primer screen sequences. All primer sequences used to amplify HPV16, HPV18 and HPV45 DNA in RPA Basic primer screens in Fig. 1.


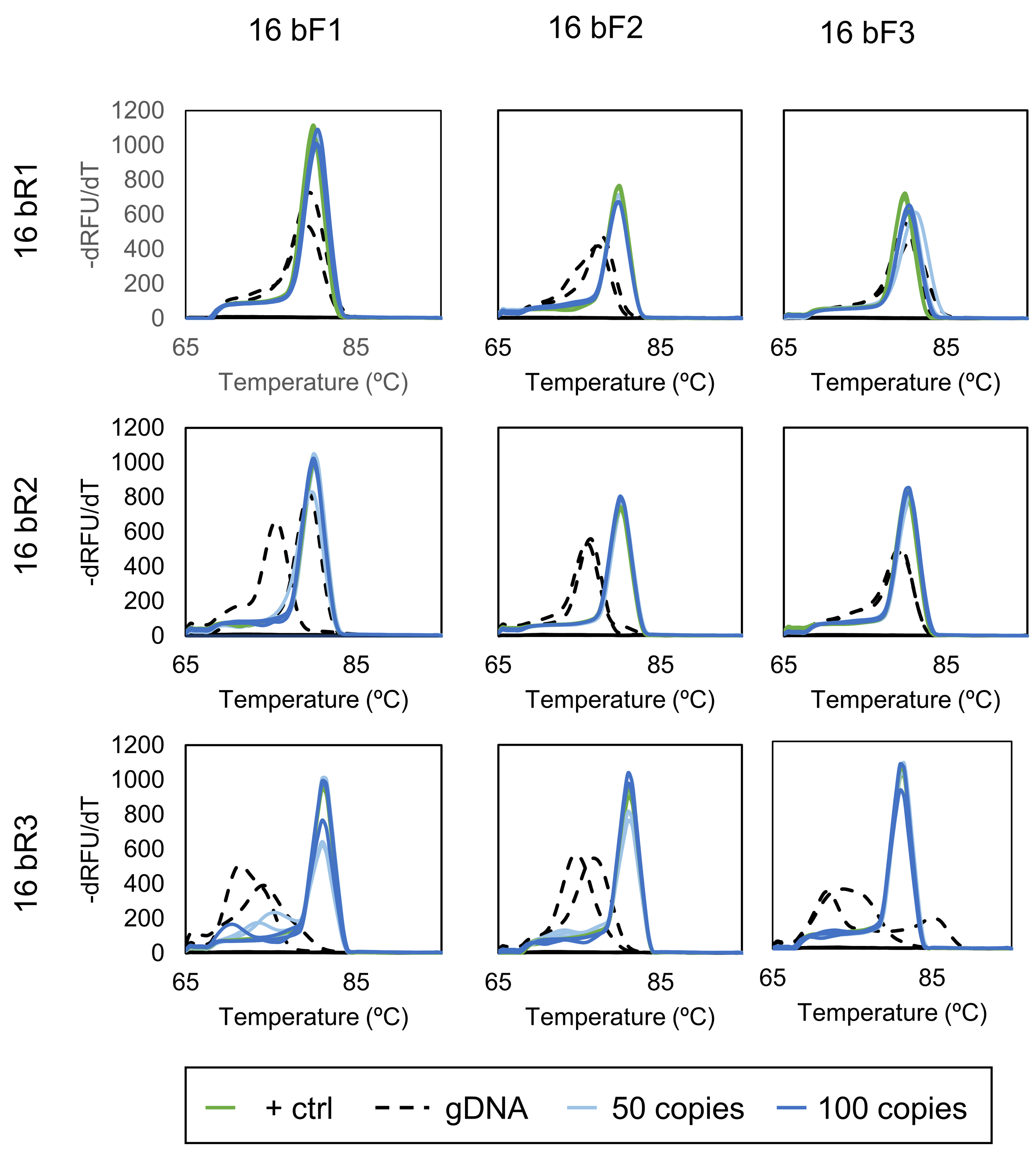


Supplementary Figure S1: Melt curve analysis of HPV16 RPA Basic primer screens. The melt curve derivatives of fluorescence change with respect to temperature are plotted for diluted RPA amplicons amplified in qPCR for each HPV16 RPA Basic primer pair screened. The melt curve derivatives of qPCR positive controls (n=3), RPA no-target gDNA controls (n=2), RPA reactions containing 50 input copies of HPV16 gBlock DNA (n=3), and RPA reactions containing 100 input copies of HPV16 gBlock DNA (n=3) are shown for each primer pair. Melt curve derivatives indicate that RPA primers form non-specific primer dimerization products in the absence of HPV16 target. In addition, non-specific product formation is seen for one RPA replicate containing 50 input copies for primer pairs 16 bF3/16 bR1 and 16 bF1/16 bR2.


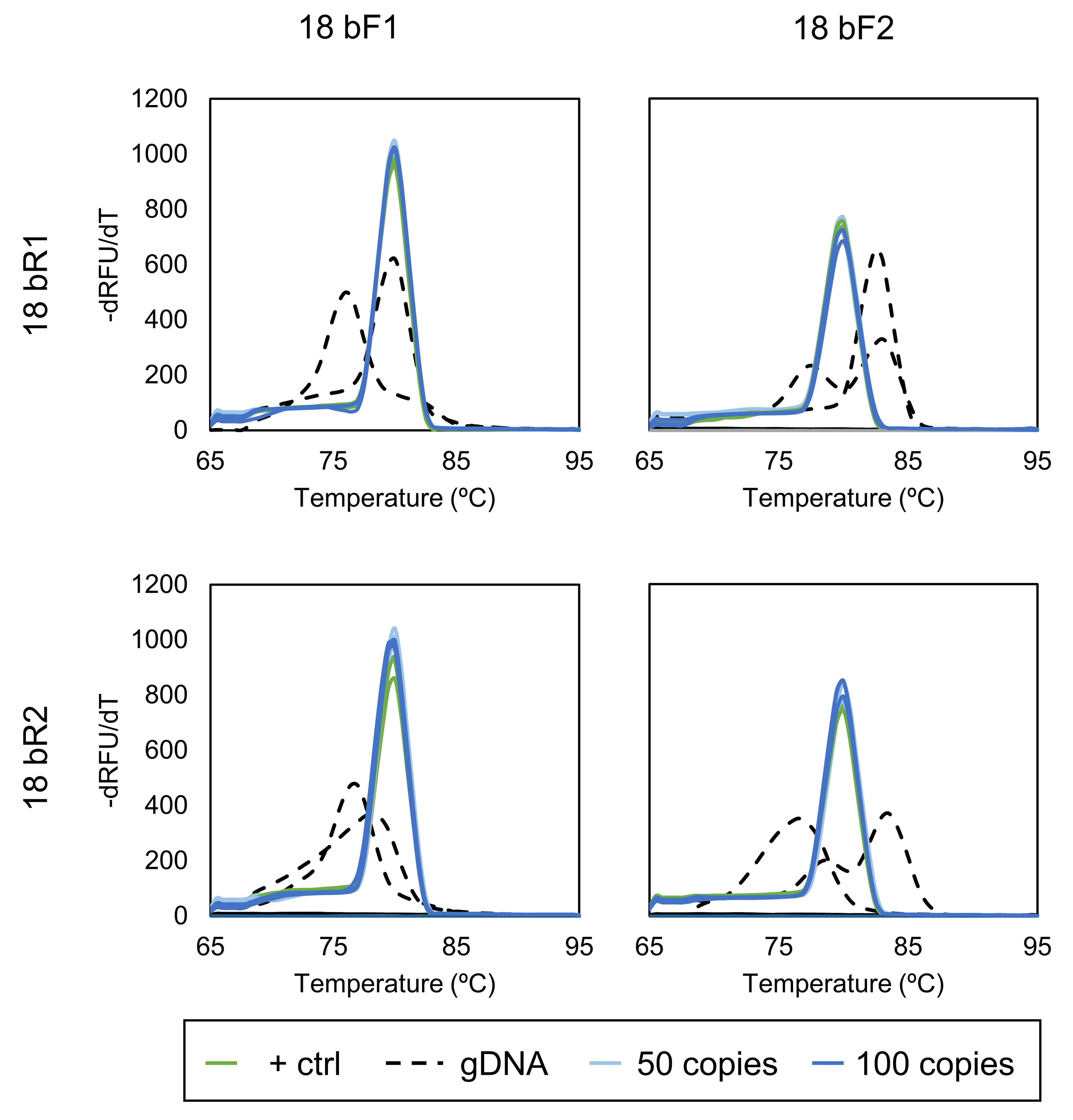


Supplementary Figure S2: Melt curve analysis of HPV18 RPA Basic primer screens. Melt curve derivatives with respect to temperature are plotted for diluted RPA amplicons amplified in qPCR for each HPV18 RPA Basic primer pair screened. The melt curve derivatives of qPCR positive controls (n=3), RPA no-target gDNA controls (n=2), RPA reactions containing 50 input copies of HPV18 gBlock DNA (n=3), and RPA reactions containing 100 input copies of HPV18 gBlock DNA (n=3) are shown for each primer pair. Melt curve analysis indicates that RPA primers form non-specific primer dimerization products in the absence of HPV18 target, but all RPA reactions containing 50 and 100 input copies of target HPV18 DNA form specific amplification products.


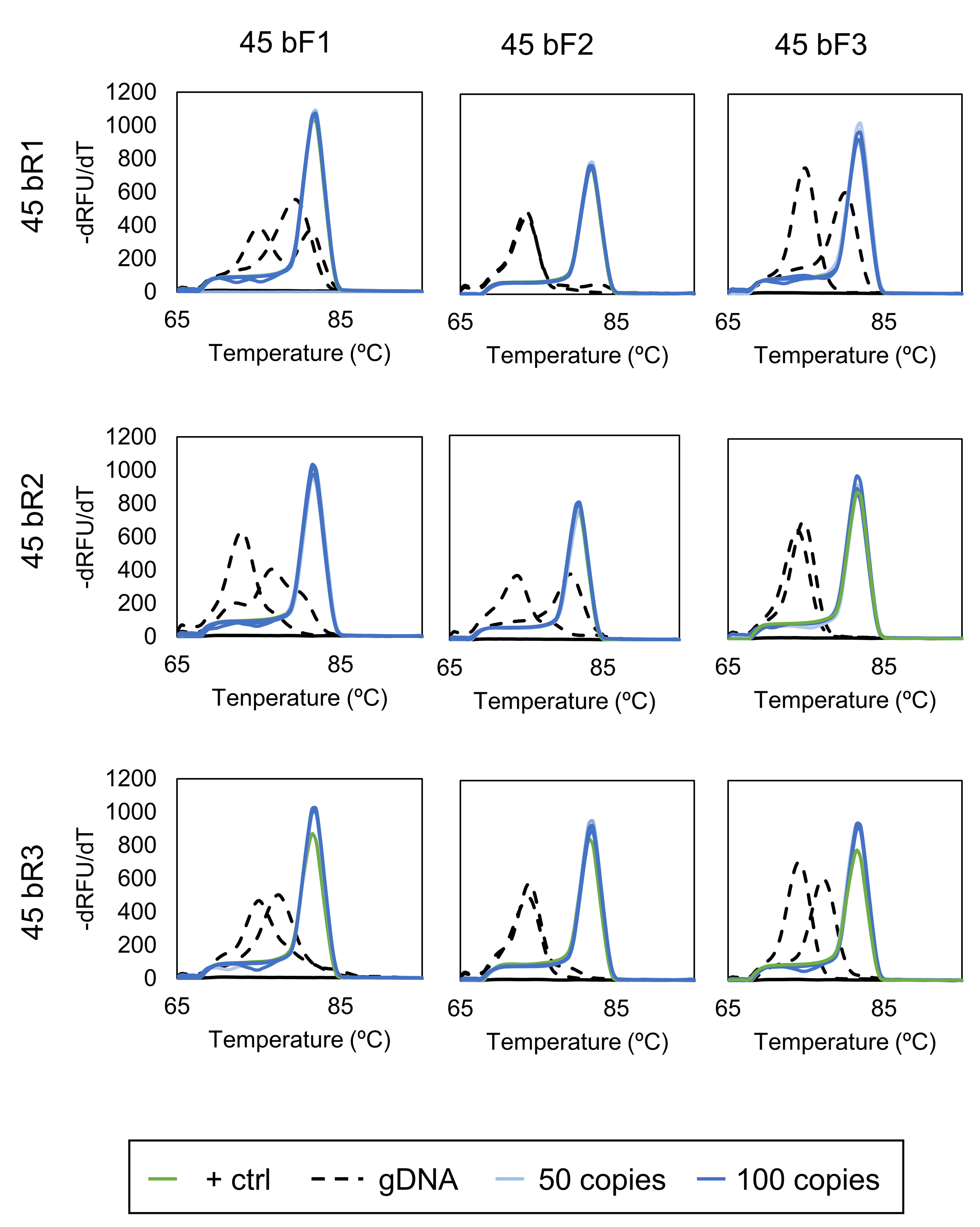


Supplementary Figure S3: Melt curve analysis of HPV45 RPA Basic primer screens. Melt curve derivatives are plotted for diluted RPA amplicons amplified in qPCR. The melt curve derivatives of qPCR positive controls (n=3), RPA no-target gDNA controls (n=2), RPA reactions containing 50 input copies of HPV45 gBlock DNA (n=3), and RPA reactions containing 100 input copies of HPV45 gBlock DNA (n=3) are shown for each primer pair. Melt curve analysis indicates that RPA primers form non-specific primer dimerization products in the absence of HPV45 target. In contrast, only specific amplification products are formed in RPA reactions containing 50 and 100 copies of target HPV45 DNA.


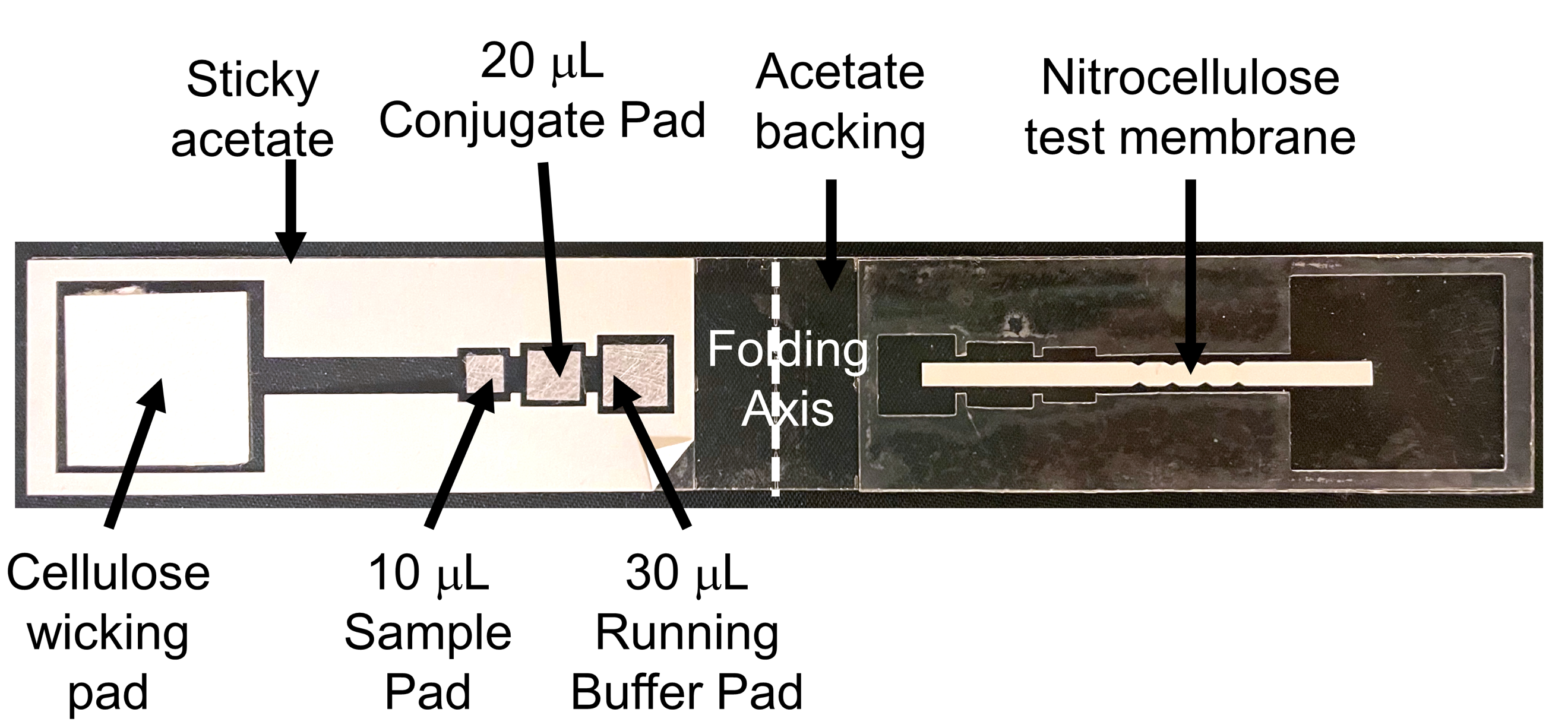


1 cm

Supplementary Figure S4: Lateral flow device for detection of tailed amplicons. The lateral flow assay to detect tailed amplicons consists of a sticky adhesive backing holding a nitrocellulose membrane with printed capture oligonucleotides and control antibody, a cellulose wicking pad, and three glass fiber pads for reagents. Liquid reagents are added to their respective pads, the sticky acetate is peeled, and the device is folded to initiate flow to deliver reagents sequentially across the test and control lines.

| Primer Name | Primer Sequence (5’ 🡪 3’) |
| --- | --- |
| 16 tP1 | ATTATCAACGAGACC/iSpC3/CACCTACATTGCATGAAT ATATGTTAGATT |
| 16 tP2 | GCTCAGTTTTGTTGT/iSpC3/CACCTACATTGCATGAAT ATATGTTAGATT |
| 16 tP3 | CTTTTGAGATTACTC/iSpC3/CACCTACATTGCATGAAT ATATGTTAGATT |
| 16 tP4 | ATCCAGATAGCGAAA/iSpC3/TAGTGTGCCCATTAACAG GTCTTCCAAAGT |
| 18 tP1 | AAACTATTCACGGCA/iSpC3/TACTAGCTCAATTCTGGC TTCACACTTACA |
| 18 tP2 | TTCTGTTACCACTAA/iSpC3/TACTAGCTCAATTCTGGC TTCACACTTACA |
| 18 tP3 | ACAGTTGTTGTCTAC/iSpC3/TACTAGCTCAATTCTGGC TTCACACTTACA |
| 18 tP4 | AAGCGTTATCTTCTT/iSpC3/ATTGCATTTAGAGCCCCA AAATGAAATTCC |
| 45 tP1 | TTGGTATTTACACAC/iSpC3/TGCATTTGGAACCTCAGA ATGAATTAGATC |
| 45 tP2 | GTCGTTGTATCATCA/iSpC3/TCTCTACTGTAAGCTCAA TTCTGCCGTCAC |
| 45 tP3 | AGATAGTTCTTTAGT/iSpC3/TCTCTACTGTAAGCTCAA TTCTGCCGTCAC |
| 45 tP4 | TAGAATACCAGTAGT/iSpC3/TGCATTTGGAACCTCAGA ATGAATTAGATC |

Supplementary Table S3: RPA tailed primer screen sequences. All tailed primer sequences used to amplify HPV16, HPV18, and HPV45 DNA in RPA tailed primer screens in Fig. 2.

| Primer Name | Corresponding Capture Probe Sequence (5’ 🡪 3’) |
| --- | --- |
| 16 tP1 | GGTCTCGTTGATAAT GGTCTCGTTGATAAT  GGTCTCGTTGATAAT GGTCTCGTTGATAAT |
| 16 tP2 | ACAACAAAACTGAGC ACAACAAAACTGAGC  ACAACAAAACTGAGC ACAACAAAACTGAGC |
| 16 tP3 | GAGTAATCTCAAAAG GAGTAATCTCAAAAG  GAGTAATCTCAAAAG GAGTAATCTCAAAAG |
| 16 tP4 | TTTCGCTATCTGGAT TTTCGCTATCTGGAT  TTTCGCTATCTGGAT TTTCGCTATCTGGAT |
| 18 tP1 | TGCCGTGAATAGTTT TGCCGTGAATAGTTT  TGCCGTGAATAGTTT TGCCGTGAATAGTTT |
| 18 tP2 | TTAGTGGTAACAGAA TTAGTGGTAACAGAA  TTAGTGGTAACAGAA TTAGTGGTAACAGAA |
| 18 tP3 | GTAGACAACAACTGT GTAGACAACAACTGT  GTAGACAACAACTGT GTAGACAACAACTGT |
| 18 tP4 | AAGAAGATAACGCTT AAGAAGATAACGCTT  AAGAAGATAACGCTT AAGAAGATAACGCTT |
| 45 tP1 | GTGTGTAAATACCAA GTGTGTAAATACCAA  GTGTGTAAATACCAA GTGTGTAAATACCAA |
| 45 tP2 | TGATGATACAACGAC TGATGATACAACGAC  TGATGATACAACGAC TGATGATACAACGAC |
| 45 tP3 | ACTAAAGAACTATCT ACTAAAGAACTATCT  ACTAAAGAACTATCT ACTAAAGAACTATCT |
| 45 tP4 | ACTACTGGTATTCTA ACTACTGGTATTCTA  ACTACTGGTATTCTA ACTACTGGTATTCTA |

Supplementary Table S4: RPA tailed primer capture probes. All capture probe sequences printed at the test lines to capture tailed primer products generated in RPA tailed primer screens (Fig. 2).


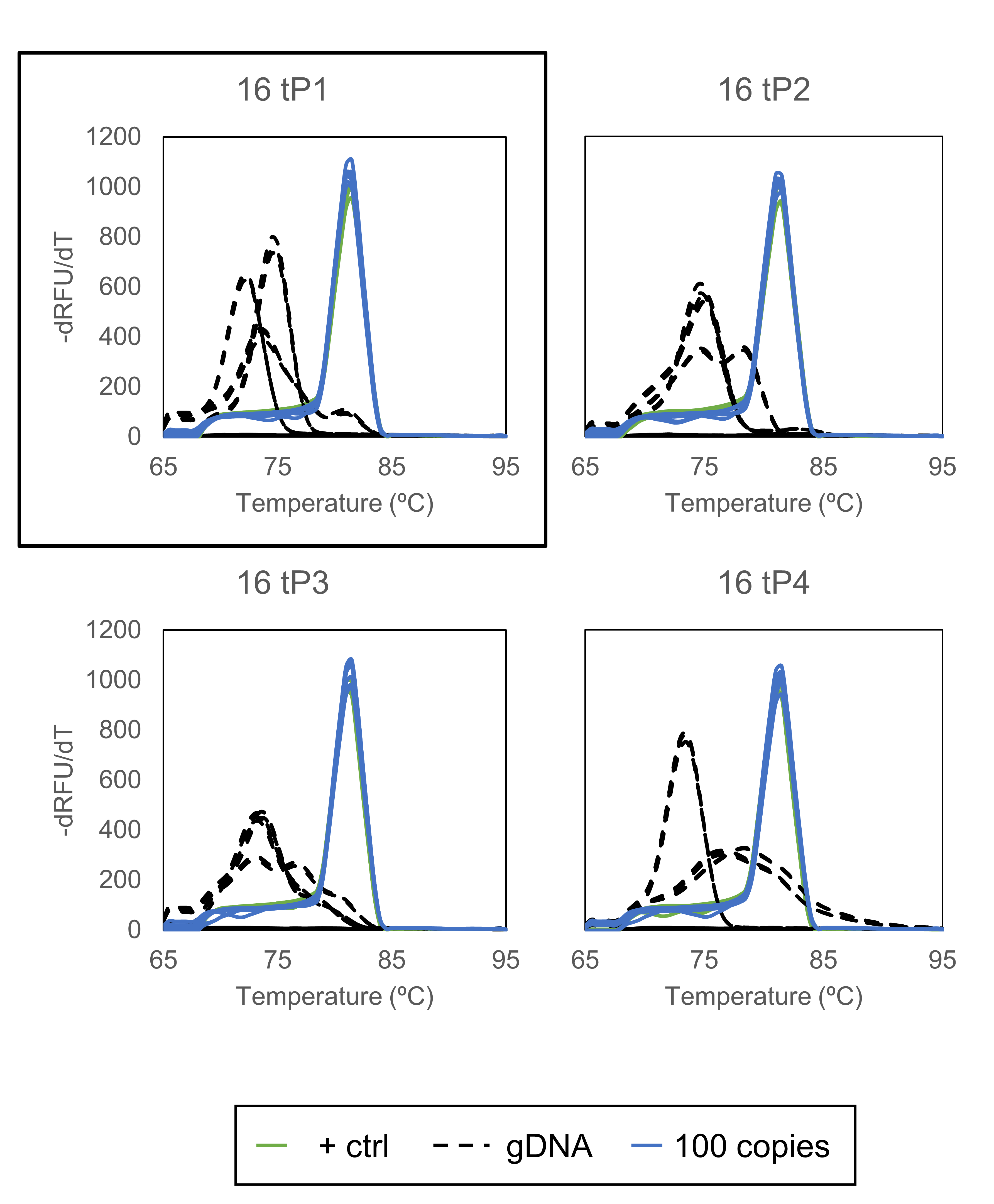


Supplementary Figure S5: Melt curve analysis of HPV16 RPA tailed primer screen. Melt curve derivatives for diluted RPA reactions containing no-target gDNA (3 RPA replicates x 3 technical replicates) and 100 input copies of HPV16 gBlock DNA (3 RPA replicates x 3 technical replicates) are plotted for each tailed primer pair screened against the qPCR positive control. Melt curves indicate that positive visual signal formed on lateral flow strips from no-target gDNA controls in Fig. 2 are a result from non-specific primer dimerization products. The best-performing tailed primer pair (16 tP1) is outlined.


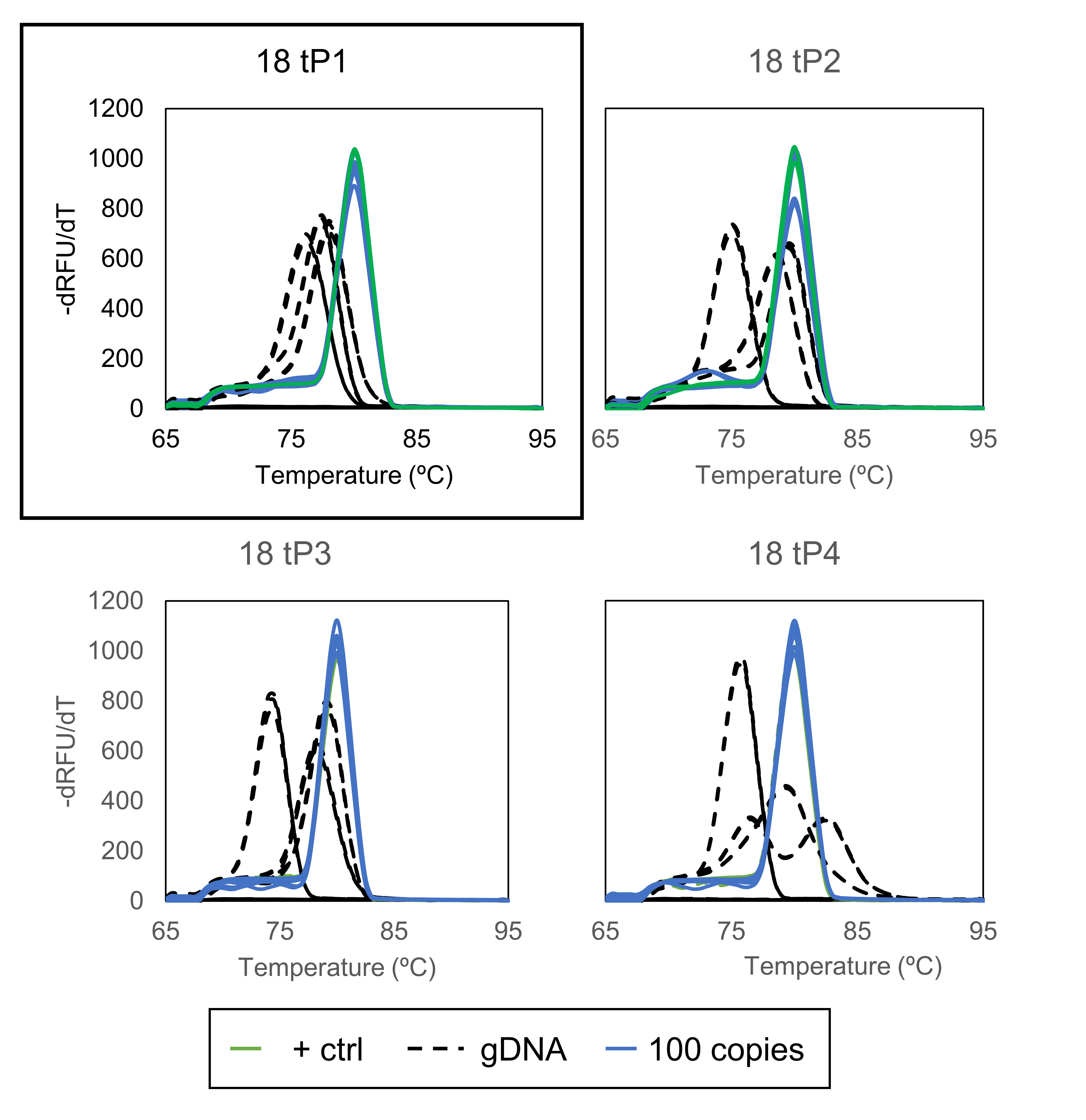


Supplementary Figure S6: Melt curve analysis from HPV18 RPA tailed primer screen. Melt curve derivatives for diluted RPA reactions containing no-target gDNA (3 RPA replicates x 3 technical replicates) and 100 input copies of HPV18 gBlock DNA (3 RPA replicates x 3 technical replicates) are plotted for each tailed primer pair screened against the qPCR positive control. Melt curves indicate that positive visual signal formed on lateral flow strips from no-target gDNA controls in Fig. 2 are a result from non-specific primer dimerization products. The best-performing tailed primer pair (18 tP1) is outlined.


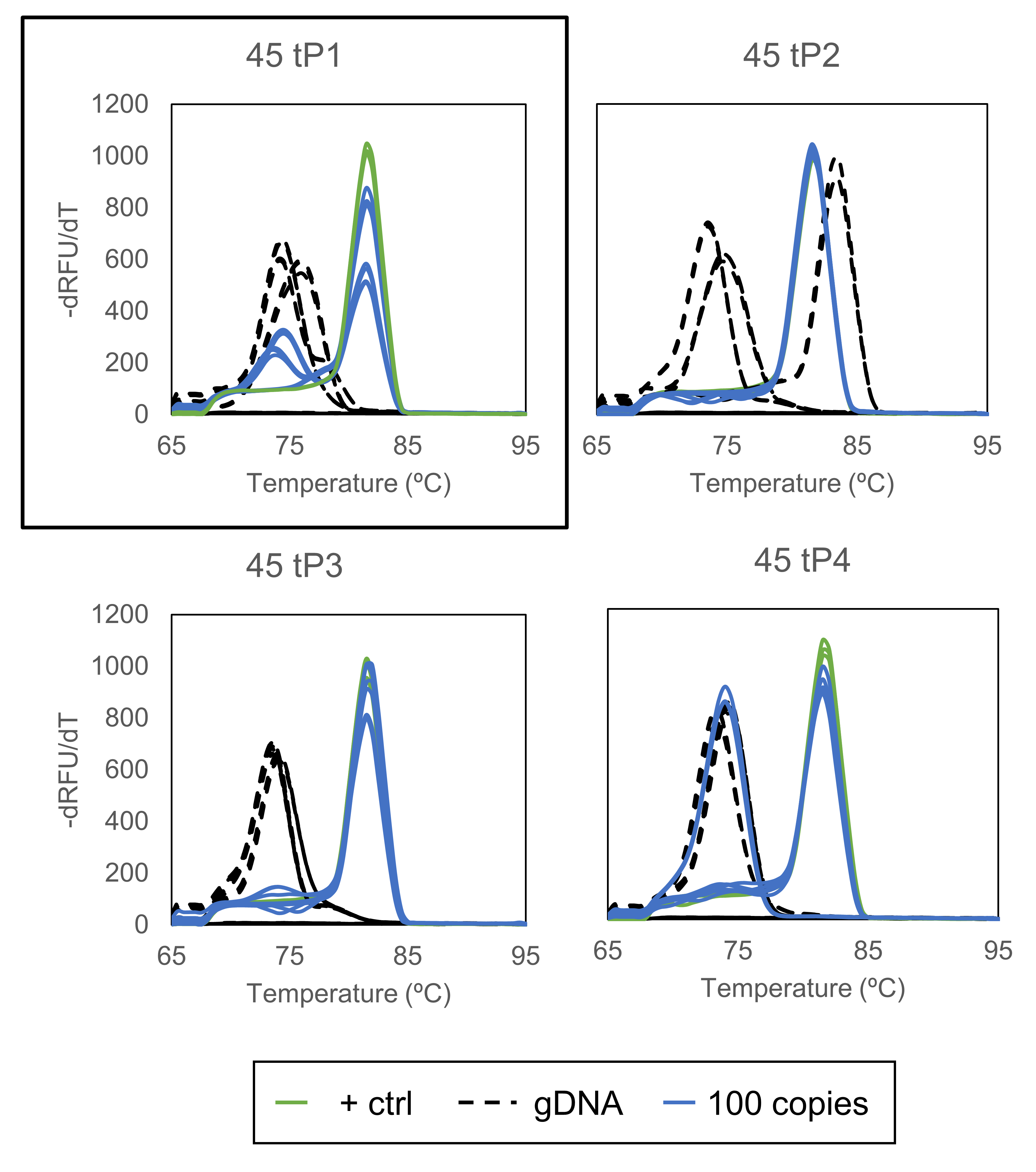


Supplementary Figure S7: Melt curves analysis of HPV45 RPA tailed primer screen. Melt curve derivatives for diluted RPA reactions containing no-target gDNA (3 RPA replicates x 3 technical replicates) and 100 input copies of HPV45 gBlock DNA (3 RPA replicates x 3 technical replicates) are plotted for each tailed primer pair screened against the qPCR positive control. Melt curves indicate that positive visual signal formed on lateral flow strips from no-target gDNA controls in Fig. 2 are a result from non-specific primer dimerization products. The best-performing tailed primer pair (45 tP1) is outlined.

## NATflow Insert Flow Validation

To validate that the insert effectively delivered fluid from all three chambers across the test zones of the lateral flow strip, NATflow cartridges were run with and without the insert using red, blue, and yellow food coloring (Supplementary Fig. S8). PCR tubes contained 10 μL of red, yellow, and blue food coloring, either mixed in a PCR-tube, or delivered to each chamber of the PCR insert within a PCR-tube, and then eluted in the NATflow cartridge in triplicate. Cartridges were videoed with a mobile phone (iPhone 12) at 30 frames per second for ten minutes after fluid flow initiation.

Using ImageJ, videos were white balanced to correct for lighting artifacts using a white balance macro from https://github.com/pmascalchi/ImageJ_Auto-white-balance-correction/. Frames were analyzed every 60s after an initial 30s delay to allow the fluid front to reach the cartridge reading window. The average vertical red, green, and blue pixel intensities at the center of the cartridge reading window were measured using the RGB profile macro on ImageJ. A two-way repeated measures ANOVA with post-hoc Bonferroni’s multiple comparison test at the 0.05 significance level was used to evaluate differences in red, green, and blue pixel intensities across time with and without the insert. There were no statistically significant differences in the red, green, or blue pixel intensities between cartridges run with and without the insert, indicating that the insert delivers fluid from all three wells of the insert across the test zone of the nitrocellulose.


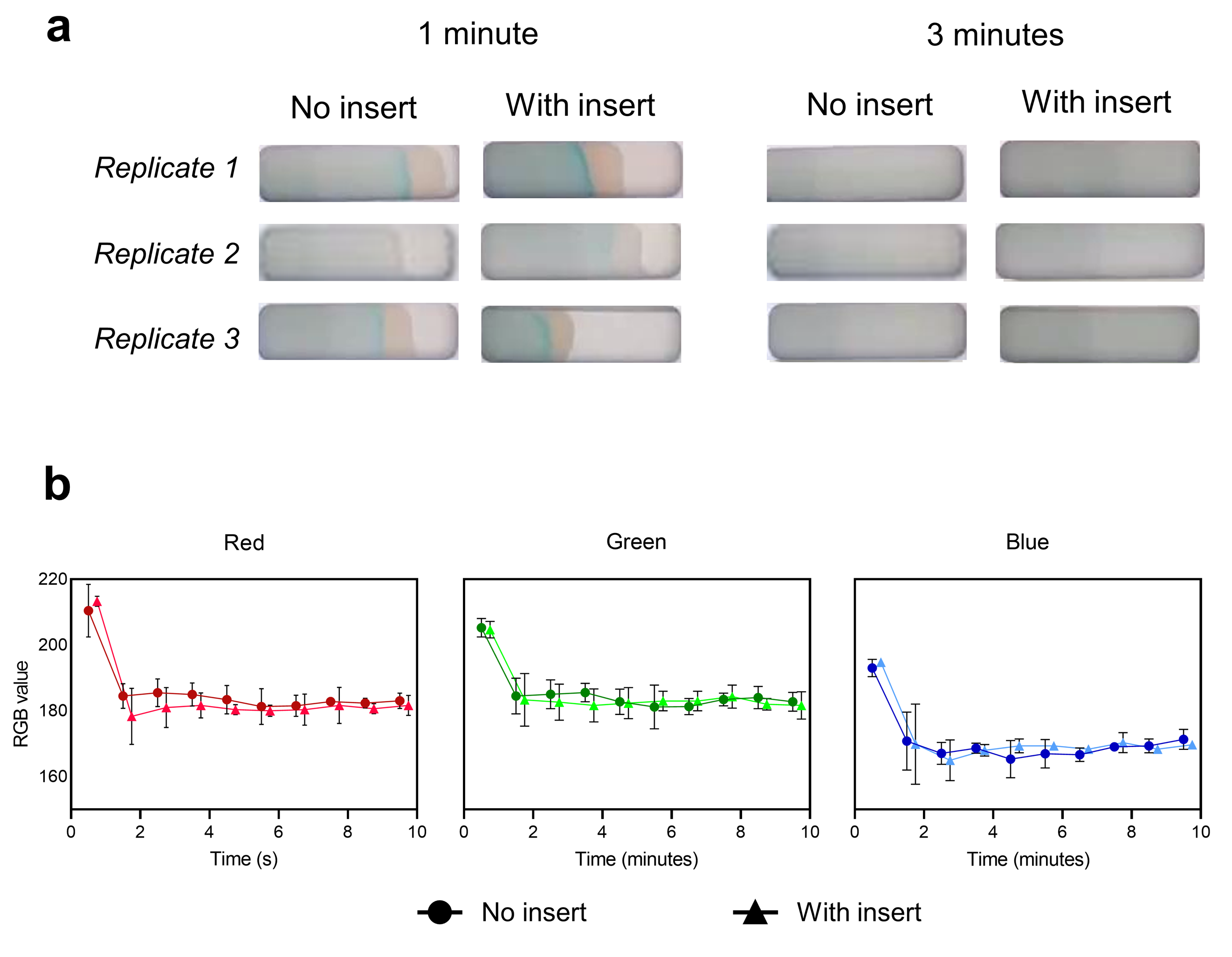


Supplementary Figure S8: NATflow flow validation with PCR insert. (A) Images of lateral flow strips run with red, blue, and yellow food coloring inside the NATflow cartridge with and without the insert after one and three minutes of flow. (B) Temporal analysis of signal intensity on lateral flow strips (n=3) with standard deviations; data sets from replicates staggered by 15 seconds for better visualization. No statistically significant differences between the red, green, and blue pixel values were seen at any time point, indicating the insert effectively delivers fluid from all three chambers to the lateral flow strip. Significance determined at the 0.05 significance level using a two-way repeated measures ANOVA with post-hoc Bonferroni multiple comparisons test.

## NATflow Insert Temperature Optimization

We wanted to ensure that reactions run within the PCR insert on the NATflow heater reached the optimal reaction temperature of 39 ºC. Using 10 μL of rehydration buffer (TwistDx) to simulate small volume RPA reactions, the temperature of the rehydration buffer incubated in a NATflow insert was measured over 20 minutes for NATflow heaters programmed to 39 ºC, 42 ºC, and 45 ºC. The time and temperature profiles of the rehydration buffer in the NATflow inserts at these set temperatures were then compared to that of rehydration buffer sitting at the bottom of the PCR tube on a NATflow set to 39 ºC (control; Supplementary Fig. S9). Results indicate that it was necessary to increase the temperature of the NATflow heating bay to 45 ºC to ensure that reactions incubated within the insert reached 39 ºC.


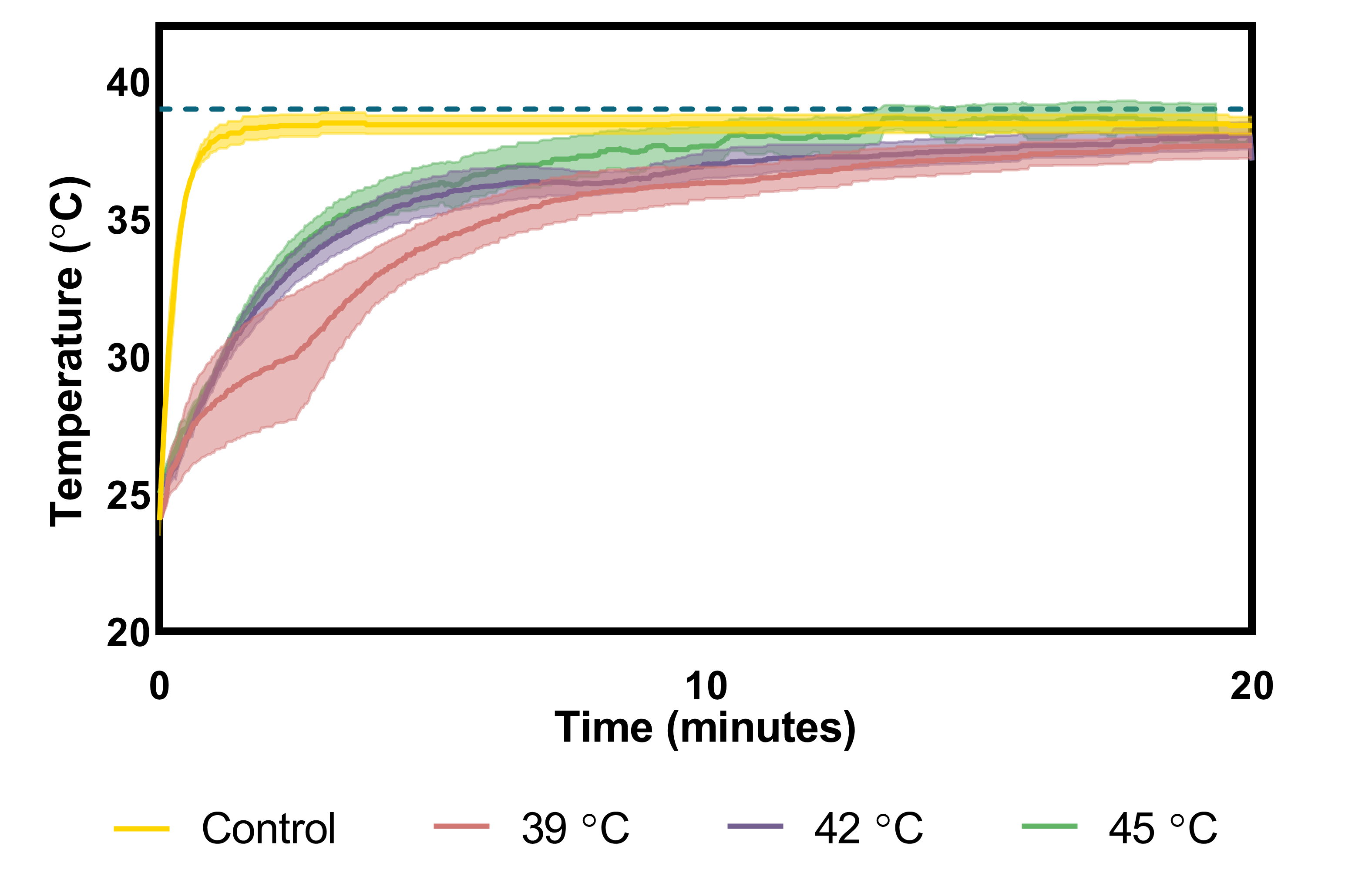


Supplementary Figure S9: Reaction temperature over time on the NATflow heater. 10 μL of rehydration buffer loaded into one of the NATflow insert wells was heated on three separate NATflow heaters with set temperatures of 39 ºC, 42 ºC, or 45 ºC. The average temperature is plotted and compared to a control condition of 10 μL of rehydration buffer heated at 39 ºC without the insert. Shading represents one standard deviation. Results indicated it was necessary to set the NATflow heating bay temperature to 45 ºC to ensure enough heat transfer to the PCR insert to reach the optimal 39 ºC reaction temperature for RPA.

| Test Component | Material | Cost per  Test (USD) |
| --- | --- | --- |
| Sample Preparation | Achromopeptidase | 0.01 |
| Amplification | RPA Basic reagents Primers and Probe | 2.56  0.01 |
|  | PCR Insert | 0.01 |
| Lateral Flow Detection | Capture Probes  Paper/Plastic Components  Elution Buffer  Gold Nanoshells  NATflow cartridge | 0.06  0.08  0.01 |
|  |  | 5.17 |
|  |  | 1.00 |
|  | Total | 8.91 |

Table S5: Estimated reagent and materials costs for the integrated HPV16, 18, 45 assay with small batch purchasing. Pre-conjugated streptavidin coated gold nanoshells are the most expensive reagent; however, that cost is expected to significantly decrease with manufacturing at-scale.
